# L'inflammation dans la Mucoviscidose

**DOI:** 10.1155/S0962935196000221

**Published:** 1996-04


Mediators of Inflammation **5**, 144–169 (1996)

### L'inflammation dans la Mucoviscidose

### Symposium international

**organisé par**

**l'Association Française de Lutte contre la Mucoviscidose,**

**l'Association Allemande Mukoviszidose E.V.**

**et le Réseau Necker Mucoviscidose**

**et sous le patronage de**

**l'Institut National de la Santé et de la Recherche Médicale**

**à**

**La Maison de la Recherche, Paris**

**14–15 juin 1996**

### Introduction

L'inflammation chronique des voies aériennes est une caractéristique physiopathologique majeure de la mucoviscidose (cystic fibrosis, CF). La colonisation bactérienne précoce et l'infection des bronches par des agents pathogènes immuables, comme *Staphylococcus aureus* et *Pseudomonas aeruginosa* favoriserait la migration massive des polynucléaires sanguins vers le poumon, la libération des médiateurs de l'inflammation et la dysrégulation de l'immunité spécifique et non spécifique. L'extension de inflammation serait largement impliquée dans la diminution de la fonction pulmonaire et la mort prématurée des patients atteints de mucoviscidose.

Au cours des dernières années, il est apparu que les mécanismes physiopathologiques qui concourent à l'inflammation dans la mucoviscidose mettent en jeu divers types de médiateurs tels que les protéinases, les dérivés réduits de l'oxygène issus des polynucléaires neutrophiles ou les cytokines pro-inflammatoires produites par les macrophages, dans un réseau complexe d'interactions et de déséquilibres avec leurs inhibiteurs sélectifs que sont les anti-protéases, les anti-oxydants et les anti-cytokines.

Le but de ce **Symposium International sur l'Inflammation dans la Mucoviscidose** est de réunir des chercheurs experts en ces divers domaines de l'inflammation et des cliniciens spécialisés dans la surveillance de la mucoviscidose afin de faire le point sur l'état actuel des connaissances et les aspects les plus cruciaux de cette importante facette de la maladie. Nous espérons qu'à l'issue de cette rencontre entre cliniciens et chercheurs intéressés à la mucoviscidose, de nouvelles stratégies thérapeutiques anti-inflammatoires plus sélectives et plus efficaces pourront être envisagées et contribuer ainsi à améliorer le pronostic vital des patients qui en sont atteints.

La présentation de **Michel Aubier, Paris, France** introduit la discussion par une description approfondie des caractéristiques de l'inflammation du poumon humain focalisée au site de l'infection pulmonaire. Puis, **Annick Clément, Paris, France** expose les études récentes montrant que l'inflammation peut devancer l'infection pulmonaire et contredire ainsi le paradigme de la précession de l'infection sur l'inflammation dans la mucoviscidose. La mise au point de nouvelles méthodes d'exploration permettant de déterminer le degré de l'inflammation chez le tout jeune enfant et de suivre son évolution serait nécessaire pour confirmer cette hypothèse de toute première importance. Il est également intéressant de souligner que l'expression et/ou la régulation des médiateurs de l'inflammation issus des macrophages et des cellules épithéliales diffère entre les patients CF et les sujets témoins.

© 1996 Rapid Science Publishers

**Melvin Berger, Cleveland, USA** fait état d'un contenu en IL-10 des cellules épithéliales bronchiques nettement plus faible chez le patient CF que chez le sujet normal et soulève le problème des relations entre le défaut de la protéine CFTR et cette altération de la production de cytokines par les cellules épithéliales. **Jean-Michel Dayer, Genève, Suisse** présente l'état des connaissances sur le réseau complexe des cytokines pro-inflammatoires et de leurs inhibiteurs. Il soulève le problème des effets combinés anti-inflammatoire et immunosuppresseur des inhibiteurs de cytokines et de l'importance de considérer cette dualité dans les perspectives de l'utilisation de ces molécules en thérapeutique. Enfin **Sophie Girod de Bentzmann, Reims, France** présente des faits montrant que la destruction et la réparation des voies aériennes secondaires à l'inflammation peuvent également favoriser l'adhésion du *Pseudomonas aeruginosa.*

Les mécanismes conduisant au recrutement des neutrophiles vers la lumière des voies aériennes dans la mucoviscidose, le relargage des enzymes lysomiaux et les stratégies de prévention sont un pôle d'intérêt majeur envisagés dans ce Symposium. En se basant sur des modèles expérimentaux d'inflammation pulmonaire, **Peter Ward, Ann Arbor, USA** discute des médiateurs mis en jeu pour le recrutement des neutrophiles dans une maladie pulmonaire. **Jay Nadel, San Francisco, USA** décrit un système complexe de signaux impliquant des composés de bactéries, l'épithélium des voies respiratoires et le polynucléaire neutrophile entraînant le chimiotactisme et la libération d'enzymes lysomiaux. **Elaine Tuomanen, New York, USA** fait état d'essais dans lesquels les anticorps contre CD18 ou les molécules qui se lient au récepteur CD18 bloquent la migration des neutrophiles. **Robert Stockley, Birmingham, UK**, résume l'état des connaissances sur les gènes des sérine-protéases des neutrophiles, comme l'élastase, la cathepsine G et la protéinase 3 et discute des mécanismes de régulation pour l'expression de l'élastase au cours de la différenciation cellulaire. **Gerd Döring, Tübingen, Allemagne** fait le point sur de récents essais thérapeutiques où l'inhibiteur de l'alpha-1-antitrypsine est administré par voie d'aérosol afin de supprimer l'activité de l'élastase produite par les neutrophiles infiltrant les voies aériennes des patients atteints de mucoviscidose.

Le stress oxydatif, conséquence du déséquilibre de la balance entre oxydants et antioxydants constitue également un chapitre important de ce Symposium. **Véronique Witko-Sarsat, Paris, France** expose l'état actuel des connaissances sur l'activation du polynucléaire neutrophile qui aboutit à la génération des dérivés réduits de l'oxygène et présente des résultats récents démontrant que cette activité est accrue chez les sujets CF homozygotes ou hétérozygotes pour la mutation de la protéine CFTR. **Franck J. Kelly, Londres, UK** démontre la présence de marqueurs de stress oxydatif et de molécules piégeurs de radicaux libres dans le plasma, l'urine et l'expectoration des sujets atteints de mucoviscidose incitant à l'utilisation d'une thérapie anti-oxydante pour réduire l'atteinte pulmonaire dans cette affection. A l'opposé, **Dieter Worlitzsch, Tübingen, Allemagne** émet l'hypothèse que l'absence de peroxyde d'hydrogène dans l'air expiré des patients CF est liée à l'effet piègeur de la myéloperoxydase et de la catalase présentes à de fortes concentrations dans les expectorations.

S'appuyant sur une large expérience clinique des médicaments anti-inflammatoires classiques, la présentation de **Michael Konstan, Cleveland, USA** démontre leur effet bénéfique limité sur les manifestations inflammatoires des voies aériennes. La thérapie génique peut être abordée comme une alternative possible à la prévention de l'inflammation pulmonaire. **Catherine Figarella, Marseille, France** expose les modalités d'expression et de fonction de la protéine CFTR dans les voies aériennes. **Patricia Lemarchand, Paris, France** aborde les obstacles à la thérapie génique substitutive de la protéine CFTR défectueuse liés notamment au stress oxydatif. Dans des modèles *in vitro* **Aleksander Edelman, Paris, France** met en exergue l'effet dépresseur des cytokines et de l'Aspirine sur l'expression du gène CFTR, suggérant ainsi que l'inflammation pourrait s'opposer à l'action de la thérapie génique. **Gabriel Bellon, Lyon, France** rapporte les résultats d'un essai de thérapie génique chez 6 patients et **Claire Danel, Paris, France** présente des résultats démontrant les conséquences inflammatoires au niveau pulmonaire du transfert de gène.

En conclusion, la mucoviscidose reflète bien la face hostile du double visage de Janus, choisi par Ivan Bonta, Editeur de *Mediators of Inflammation* comme 'symbole *par excellence*' de ce journal. Nous espérons que les récentes découvertes fondamentales sur le rôle pivot de l'inflammation dans la mucoviscidose et les nouvelles stratégies thérapeutiques présentées et discutées à ce Symposium pourront avoir un impact important dans la surveillance et le traitement de l'inflammation dans la mucoviscidose et contribuer ainsi à améliorer le pronostic vital des patients qui en sont atteints.

**Béatrice Descamps-Latscha, MD, PhD**
*INSERM U25, Hôpital Necker, Paris, France*

**Gerd Döring, PhD**

*Hygiene-Institut, Tübingen, Germany*

**Pierre Galanaud, MD**

*INSERM U131, Hôpital Antoine Béclère, Clamart, France*

**Gérard Lenoir, MD**
*Hôpital Necker, Paris, France*

**Jean Navarro, MD**
*Hopital Robert Debré, Paris, France*

**Laurence Schaffar, MD, PhD**
INSERM, Paris, France

REMERCIEMENTS. Nous exprimons toute notre reconnaissance au Professeur Ivan Bonta, Editeur de *Mediators of Inflammation*, d'avoir accepté de publier les actes de ce Symposium dans une version bilingue et l'éditeur de Rapid Science Publishers qui a réalisé cette publication à titre gracieux et a accéléré la parution de ce numéro de *Mediators of Inflammation* afin que nous puissions en disposer dès l'ouverture du Symposium.

### Liste des Participants

Michel Aubier, MD, Paris, France; Gabriel Bellon, MD, Lyon, France; Melvin Berger, MD, Cleveland, USA; Annick Clément, MD, Paris, France; Claire Danel, MD, Paris, France; Jean-Michel Dayer, MD, Geneva, Switzerland; Béatrice Descamps-Latscha, MD, Paris, France; Gerd Döring, PhD, Tübingen, Germany; Daniel Dusser, MD, Paris, France; Aleksander Edelman, PhD, Paris, France; Catherine Figarella, MD, Marseille, France; Pierre Galanaud, MD, Clamart, France; Eva Giesen, PhD, Paris, France; Sophie Girod de Bentzmann, PhD, Reims, France; Michel Goossens, MD, Créteil, France; Frank J. Kelly, MD, London, UK; Michael Konstan, MD, Cleveland, USA; Patricia Lemarchand, MD, Paris, France; Gérard Lenoir, MD, Paris, France; Jay Nadel, MD, San Francisco, USA; Jean Navarro, MD, Paris, France; Laurence Schaffar, MD, Paris, France; Robert A. Stockley, MD, Birmingham, UK; Elaine Tuomanen, MD, New York, USA; Peter Ward, MD, Ann Arbor, USA; Véronique Witko-Sarsat, PhD, Paris, France; Dieter Worlitzsch, MD, Tübingen, Germany.

### Résumés

### L'inflammation pulmonaire focalisée au site de l'infection

*Michel Aubier*
Service de Pneumologie, Unité INSERM 408, Hôpital Bichat, 46 rue Henri Huchard, 75018 Paris, France

Plusieurs mécanismes de défense sont localisés le long des voies respiratoires supérieures normales et empêchent, les bactéries d'atteindre les alvéoles. De plus, les bactéries qui parviennent jusqu'aux alvéoles sont généralement phagocytées et détruites par des macrophages résidents.[1] Lorsque les mécanismes d'élimination sont débordés, une réponse complexe se développe. L'invasion d'un tissu périphérique par une bactérie pathogène entraîne une réponse inflammatoire accompagnée d'une activation de macrophages avoisinants par des produits bactériens tels que l'endotoxine et des composants des parois. Le stade initial de l'infection peut être caractérisé par la production de signaux multiples, parmi lesquels on retrouve une cascade de médiateurs endogènes de l'hôte déclenchée par les produits bactériens. Parmi ces médiateurs, les cytokines et plus particulièrement le facteur nécrosant des tumeurs-α (TNF-α), l'interleukine-1β (IL-1β), l'interleukine-6 (IL-6) et l'IL-8 semblent intervenir dans les réponses de l'hôte à l'infection bactérienne. Ces cytokines sont impliquées dans la réponse à l'infection, et sont à l'origine de l'activation des cellules productrices d'anticorps spécifiques. Après l'activation des cellules endothéliales, les cytokines sont également impliquées dans le recrutement et l'activation des monocytes et des neutrophiles vers les sites infectés.

Bien qu'une production excessive de cytokines au cours d'une infection ait de nombreux effets délétères pouvant entraîner la mort,[2] plusieurs modèles animaux suggèrent un rôle bénéfique des cytokines dans la résistance naturelle de l'hôte à l'infection locale. Les cytokines produites localement contribueraient ainsi à l'éradication du pathogène invasif[3]. En effet, certaines cytokines auraient plus d'effets locaux dans leur tissu d'origine que d'effets systémiques.[4–6]

A cet égard, plusieurs études chez l'homme et chez l'animal ont démontré des différences significatives dans la réponse inflammatoire obtenue dans des modèles d'infection intravasculaire et celle obtenue au cours d'une infection bactérienne localisée, telle que la méningite.[4,5] Néanmoins, la réponse inflammatoire *in situ* qui se développe dans le poumon humain au cours d'une infection bactérienne localisée, demeure largement méconnue.

La réponse inflammatoire *in situ* qui se développe dans le poumon humain lors d'une infection bactérienne localisée a été observée au cours de deux études récentes[7,8] chez 15 patients ayant une CAP (community-acquired pneumonia) unilatérale. La réponse locale dans le poumon impliqué a été comparée avec celle du poumon controlatéral, non impliqué ainsi qu'avec la réponse systémique sanguine. Huit volontaires sains ont servi de sujets témoins. Les concentrations en facteur nécrosant des tumeurs-α (TNF-α), d'interleukine-1β (IL-1β), d'interleukine-6 (IL-6) et d'IL-8 ont été mesurées par ELISA dans les fluides ($n = 15$) de lavages bronchoalvéolaires (BAL), le sérum ($n = 15$), et les surnageants de cultures de macrophages et monocytes alvéolaires ($n = 8$). Les concentrations de TNF-α, IL-1β, IL-6 et IL-8 dans les fluides BAL étaient significativement plus élevées dans le poumon atteint que dans le poumon sain ($p \leqslant 0.01$) ou chez les sujets sains (respectivement $p \leqslant 0.02$, $p \leqslant 0.01$, $p \leqslant 0.001$, $p \leqslant 0.0001$). Les concentrations sériques d'IL-6 étaient plus élevées chez les patients que chez les sujets témoins, tandis que les concentrations d'IL-1β, de TNF-α et d'IL-8 ne différaient pas entre les deux groupes. Les macrophages alvéolaires situés dans le poumon atteint sécréteraient des concentrations plus élevées d'IL-1β, d'IL6 et de TNF-α ($p \leqslant 0.05$) que les macrophages du poumon sain servant de témoins. Cependant, après stimulation par le lipopolysaccharide (LPS) la production de cytokines des macrophages dans les poumons (atteint ou sain) était plus faible par rapport à celle des témoins; celle des monocytes du sang périphérique n'était pas affectée.

Ces données obtenues chez l'homme semblent démontrer que la réponse inflammatoire développée au cours d'une CAP unilatérale est focalisée à l'intérieur du poumon et est limitée au site d'infection. Bien qu'il soit admis que le TNF-α et l'IL-1β aient des effets synergiques pouvant endommager les poumons et induire une perméabilité de la paroi capillaire,[9] une limitation bénéfique de la diffusion bactérienne a également été attribuée aux cytokines.[10] La production focalisée de cytokines au niveau du poumon pendant une pneumonie unilatérale confirme l'importance du rôle joué par ces substances dans la réponse locale pulmonaire à l'infection. De même, l'absence de synthèse de cytokines suite à une stimulation au LPS pourrait résulter d'un processus homéostatique qui limiterait la réponse inflammatoire locale et protégerait ainsi le poumon des effets nocifs d'une production excessive de cytokines, qui autrement pourrait endommager les tissus, notamment les cellules épithéliales alvéolaires.

Il a été démontré que les cellules épithéliales alvéolaires de type II (cellules ATII) jouent un rôle actif dans la régulation de la réaction inflammatoire au niveau de la lumière alvéolaire. En effet, les cellules ATII ont une localisation stratégique leur permettant de moduler l'activité immunologique dans la lumière alvéolaire; les données *in vitro* et *in vivo* suggèrent que les ATII pourraient participer au réseau des cytokines intra-alvéolaires en sécrétant de l'interleukine-8 (IL-8),[11] de l'interféron,[12] de la protéine-1 chimiotactique des monocytes,[13] du facteur de croissance transformant beta (TGFβ)[14] du facteur de croissance dérivé des plaquettes (PDGF)[15,16] et du facteur de stimulation des colonies de granulocytes et de monocytes (GM-CSF),[17] et plus récemment de l'IL-6 après stimulation appropriée.[18]

Cette dernière cytokine exercerait des fonctions anti-inflammatoires. *In vitro*, à des concentrations pathologiques, l'IL-6 inhibe les réponses des cellules T via l'activation de macrophages qui sécrètent du TGF-β, une cytokine qui altère la réponse proliférative des cellules T et des thymocytes.[19] De plus, l'IL-6 régule de façon négative l'expression des gènes IL-1β et TNFα dans les monocytes humains activés,[20] module la synthèse de l'alpha-antitrypsine *in vitro* dans les phagocytes mononucléés[21] et induit un dysfonctionnement des fonctions des lymphocytes NK (natural killer).[22] *In vivo* chez le rat, de l'IL-6 intratrachéale a partiellement inhibé l'alvéolite neutrophilique induite par du LPS intratrachéal.[23]

Il apparaîtrait donc que la communication cellulaire entre des cellules immunes et non-immunes soit un processus essentiel dans l'initiation, la maintenance et la résolution de la réponse inflammatoire intra-alvéolaire. Une compréhension plus approfondie des mécanismes régulant les processus inflammatoires au niveau de la lumière alvéolaire pourrait conduire à une approche thérapeutique nouvelle et plus rationnelle chez les patients atteints d'alvéolite infectieuse ou non.

### Références

1. Sibille Y, Reynolds HY. Macrophages and polymorphonuclear neutrophils in lung defense and injury. *Am Rev Respir Dis* 1990; **141**: 471–501.
2. Waage A, Brandtzaeg P, Halstensen A, Kierulf P, Espevik T. The complex pattern of cytokines in serum from patients with meningococcal septic shock. *J Exp Med* 1989; **169**: 333–338.
3. Havell EA. Evidence that tumour necrosis factor has an important role in anti-bacterial resistance. *J Immunol* 1989; **143**: 2894–2899.
4. Rugo HS, O'Hanley P, Bishop AG, *et al.* Local cytokine production in a murine model of *Escherichia coli* pyelonephritis. *J Clin Invest* 1992; **89**: 1032–1039.
5. Waage A, Halstensen A, Shalaby R, Brandtzaeg P, Kierulf P, Espevik T. Local production of tumor necrosis factor-a, interleukin-1, and interleukin-6 in meningococcal meningitis. *J Exp Med* 1989; **170**: 1859–1867.
6. Wimott RW, Kassab JT, Kilian PL, Benjamin WR, Douglas SD, Wood RD. Increased levels of interleukin-1 bronchoalveolar washings from children with bacterial pulmonary infections. *Am Rev Respir Dis* 1990; **142**: 365–368.
7. Dehoux MS, Boutten A, Ostinelli J, *et al.* Compartmentalized cytokine production within the human lung in unilateral Pneumonia. *Am J Respir Crit Care Med* 1994; **150**: 710–716.
8. Boutten A, Dehoux MS, Seta N, *et al.* Compartmentalized IL-8 and elastase release within the human lung unilateral pneumonia. *Am J Respir Crit Care Med* 1996; **153**; 336–342.
9. Okusawa S, Gelfand JA, Ikejima T, Connoly RJ, Dinarello CA. Interleukin-1 induces a shock-like state in rabbits: synergism with tumor necrosis factor and the effect of cyclooxygenase inhibition. *J Clin Invest* 1988; **81**: 1162–1172.
10. Munoz C, Carlet J, Fitting C, Misset B, Bleriot JP, Cavaillon J. Dysregulation of in vitro cytokine production by monocytes during sepsis. *J Clin Invest* 1991; **88**: 1747–54.
11. Standiford TJ, Kundel SL, Basha MA, *et al.* Interleukin-8 gene expression by a pulmonary epithelial cell line. A model for cytokine networks in the lung. *J Clin Invest* 1990; **86**: 1945–1953.
12. Hahon N, Castranova V. Interferon production in rat type II pneumocytes and alveolar macrophages. *Exp Lung Res* 1989; **15**: 429–445.
13. Standiford TJ, Kundel SL, Phan SH, Rollins BJ, Strieter RM. Alveolar macrophage-derived cytokines induce monocyte chemoattractant protein-1 expression from human pulmonary type II-like epithelial cells. *J Biol Chem* 1991; **266**; 9912–9918.
14. Khalil N, O'Connor RN, Unruch HW, *et al.* Increased production and immuno-histochemical localization of transforming growth factor beta in idiopathic pulmonary fibrosis. *Am J Respir Cell Mol Biol* 1991; **5**: 155–162.
15. Antoniades HN, Bravo MA, Avila T, Galanopoulos T, Neville-Golden J. Platelet-derived growth factor in idiopathic pulmonary fibrosis. *J Clin Invest* 1990; **86**: 1055–1064
16. Vignaud JM, Allam M, Martinet N, Pech M, Plenat F, Martinet Y. Presence of platelet-derived growth factor in normal and fibrotic lung is specifically associated with interstitial macrophages, while both interstitial macrophages and alveolar epithelial cells express the C-sis proto-oncogène. *Am J Respir Cell Mol Biol* 1991; **5**: 531–538.
17. Tazi A, Bouchonnet F, Grandsaigne M, Boumsell L, Hance AJ, Soler P. Evidence that granulocyte-macrophage colony stimulating factor regulates the distribution and differentiated state of dendritic cells/Langherhans cells in human lung and lung cancers. *J Clin Invest* 1991; **91**: 566–576.
18. Crestani B, Cornillet P, Dehoux M, Rolland C, Guenounou M, Aubier M. Alveolar type II epihelial cells produce interleukin-6 *in vitro* and *in vivo*. Regulation by alveolar macrophage secretory products. *J Clin Invest* 1994; **94**: 731–740.
19. Zhou D, Munster A, Winchurch RA. Pathologic concentrations of interleukin-6 inhibit T cell responses via induction of activation of TGF-β. *FASEB* 1991; **5**: 2582–2585.
20. Schindler R, Mancilla J, Endres S, Ghorbani R, Clark SC, Dinarello CA. Correlations and interactions in the production of interleukin 6 (IL-6), IL-1, and tumor necrosis factor (TNF) in human blood mononuclear cells: IL-6 suppresses IL-1 and TNF. *Blood* 1990; **75**: 40–47.
21. Perlmutter DH, May LT, Sehgal PB. Interferon β2/interleukin-6 modulates synthesis of α1-antitrypsin in human mononuclear phagocytes and in human hepatoma cells. *J Clin Invest* 1989; **84**: 138–144.
22. Tanner J, Tosato G. Impairment of natural killer functions by interleukin-6 increases lymphoblastoid cell tumorigenicity in athymic mice. *J Clin Invest* 1991; **88**: 239–247.
23. Ulich TR, Yin S, Guo K, Yi ES, Remick D, Del Castillo J. Intratracheal injection of endotoxin and cytokines. II. Interleukin-6 and transforming growth factor beta inhibit acute inflammation. *Am J Pathol* 1991; **138**: 1097–1101.

### Mécanismes de l'inflammation dans la mucoviscidose

*Annick Clément*

Département de Pneumologie Pédiatrique de l'Hôpital Armand Trousseau, Paris, France

Les manifestations respiratoires dans la mucoviscidose sont liées à une infection bronchopulmonaire chronique entrainant une insuffisance respiratoire de type obstructive. La colonisation de l'arbre respiratoire par des agents bactériens tels que le *pseudomonas aeruginosa* a été initialement considérée comme l'élément déterminant dans l'initiation et le développement des processus inflammatoires. Cependant, it est vraisemblable que la cascade d'événements associée à la progression de la maladie implique tout d'abord le déclenchement d'une inflammation pulmonaire et secondairement l'apparition d'une infection des voies respiratoires qui serait ainsi la conséquence de l'agression inflammatoire initiale. Cette conception actuelle de la physiopathologie de la maladie repose sur les résultats d'études récentes documentant la présence précoce d'une composante inflammatoire. Ainsi, l'analyse de liquides de lavage bronchoalvéolaire de très jeunes enfants atteints de mucoviscidose a permis de mettre en évidence la présence de marqueurs d'inflammation, avec en particulier une augmentation du nombre des neutrophiles, une activité élastasique accrue, et un taux important d'IL-8. Ces facteurs étaient présents alors même que les différentes investigations microbiologiques ne retrouvaient pas d'agents bactériens, de virus respiratoires, ni d'agents mycotiques.

Ces données récentes sont importantes pour deux raisons. En premier lieu, la compréhension des mécanismes associés à l'initiation de la réponse inflammatoire chez les très jeunes enfants atteints de mucoviscidose apparait comme un élément tout à fait déterminant pour l'orientation et le développement des nouvelles stratégies thérapeutiques. Plusieurs hypothèses concernant ces mécanismes peuvent être actuellement discutées. Les cellules essentiellement impliquées dans la mise en place d'un processus inflammatoire quelqu'en soit la cause sont les macrophages locaux. Il est actuellement établi que dans la mucoviscidose, les macrophages des voies respiratoires supérieures et inférieures sont une source importante d'IL-8. La raison d'une production importante d'IL-8 par ces cellules est discutée. Elle peut être la conséquence d'une stimulation excessive par des facteurs présents dans l'environnement des cellules, tel que le mucus anormal qui s'accumule dans les voies respiratoires. Elle peut également être expliquée par une altération fonctionnelle des macrophages. En effet, le gène CFTR (cystic fibrosis transmembrane conductance regulator gene) est exprimé dans les macrophages et il semble jouer un rôle dans le contrôle de certaines activités de ces cellules, en particulier dans la production et la secrétion de différentes cytokines. Un autre élément clé dans la pathogénie de la réponse inflammatoire dans la mucoviscidose est représenté par l'épithélium qui tapisse les voies respiratoires. Les cellules qui composent cet épithélium sont en effet connues pour leur capacité à produire un large éventail de médiateurs de l'inflammation. Elles sont partie intégrante du système immun local. Dans de nombreuses pathologies respiratoires, une altération de la structure et de la fonction de ces cellules a été bien documentée et est considérée comme un facteur déterminant dans la pathogénie de ces atteintes. Il en est de même dans la mucoviscidose. Les cellules de l'épithélium respiratoire peuvent, en effet, participer à la destruction progressive du tissu pulmonaire par le biais d'interactions avec les agents pathogènes, ainsi que par l'interaction avec les autres cellules présentes dans les voies respiratoires. Ces interactions sont très certainement impliquées dans l'augmentation de la production des médiateurs de l'inflammation. Cependant, les mécanismes associés à cette libération excessive, ainsi que le rôle du gène CFTR dans cette dysrégulation restent à préciser.

Le second point important concerne la nécessité de définir des marqueurs précoces d'inflammation pulmonaire chez les très jeunes enfants porteurs de mucoviscidose. En effet, si l'inflammation représente un facteur initial déterminant, il est essentiel de pouvoir la mettre en évidence le plus rapidement possible pour orienter le plus efficacement les thérapeutiques adaptées et espérer ainsi ralentir la progression de la maladie. Dans cette optique, le lavage bronchoalvéolaire est certainement intéressant, mais son utilisation est limitée car sa répétition chez le très jeune enfant est difficilement réalisable. De nouvelles approches d'évaluation de l'inflammation pulmonaire doivent donc être étudiées. Elles incluent, en particulier, l'analyse du produit d'expectoration et du produit d'aspiration des voies respiratoires hautes.

### Modèles expérimentaux d'inflammation pulmonaire

*Peter A. Ward*
Department of Pathology, The University of Michigan Medical School, Ann Arbor, Michigan, USA

Les modèles animaux d'inflammation ont procuré d'importantes informations sur les mécanismes de déclenchement des réponses cellulaires inflammatoires et sur la façon dont les lésions tissulaires se développent. Le système expérimental le mieux caractérisé de lésion inflammatoire aiguë pulmonaire du rat, découle de l'utilisation du modèle de complexes immuns à IgG dans lequel le dépôt alvéolaire de complexes immuns conduit à l'activation *in situ* du complément. Ces immuncomplexes, associés à des produits du complément, activent les macrophages pulmonaires pour induire la production précoce des cytokines TNF-$\alpha$ et IL1.

Parallèlement, ces cellules génèrent la chémokine C-C, MIP 1$\alpha$, qui semble avoir des effets de rétroaction positive autocrine, augmentant la production macrophagique de TNF-$\alpha$ et d'IL1. Le principal effet de ces deux cytokines est d'induire l'hyperexpression des molécules membranaires ICAM et E-selectine des vaisseaux pulmonaires. Par l'intermédiaire d'une série des réactions favorisant les interactions adhésives, ces molécules d'adhésion facilitent l'entrée des neutrophiles dans le compartiment alvéolaire. Le recrutement des neutrophiles fait intervenir pour le compartiment vasculaire les molécules E-sélectine, ICAM-1 et CD11a/CD18 et pour le compartiment aérien, les molécules CD11b/CD18 et ICAM-1.

L'influx chimiotactique des neutrophiles dans le poumon est médié au moins en partie par une chémokine C-X-C: MIP-2. Les lésions des parois alvéolaires et vasculaires sont induites par le monoxyde d'azote (NO) généré par la NO synthase inductible (iNOS) qui a deux sources cellulaires dans le poumon: les pneumocytes de type II et les macrophages. L'induction de iNOS est régulée par trois cytokines: le TNF-$\alpha$, l'IL-1 et l'interféron-$\gamma$.

Les lésions de la matrice pulmonaire surviennent à la suite du relargage par les macrophages et les neutrophiles de sérine-protéases et de métalloprotéases. Ces réactions inflammatoires pulmonaires sont auto-limitées, apparemment en raison de la secrétion concomitante d'IL-10 qui supprime la production de TNF-$\alpha$ et d'IL-10 elle-même par les macrophages. La production endogène d'IL-10 a été démontrée par l'apparition d'ARN messager et de protéine IL-10 dans le poumon dans les suites d'un dépôt d'immuncomplexes. Le blocage de l'IL-10 endogène conduit à une augmentation significative des lésions pulmonaires.

Ces résultats démontrent que les réactions inflammatoires pulmonaires sont induites par plusieurs cytokines proinflammatoires et régulées par au moins une cytokine anti-inflammatoire, l'IL-10.

### Processus de lésion et réparation de l'épithélium respiratoire

*Sophie Girod de Bentzmann*
INSERM U314, CHR Maison Blanche, 45, rue Cognacq Jay, 51092 Reims Cédex, France

Dans le tractus respiratoire humain normal, l'épithélium de surface de type pseudostratifié est protégé des aérocontaminants comme les virus, les bactéries ou les particules par la double protection d'une couche de mucus gel et d'un mécanisme de clairance mucociliaire (CMC) actionné par le battement ciliaire.

Dans des conditions pathologiques, comme dans la mucoviscidose, la CMC est défectueuse, en particulier à cause d'une hypersécretion et d'une hyperviscosité du mucus respiratoire. Dans la mucoviscidose, la diminution de la CMC associée à une inflammation chronique permet l'instauration de conditions propices pour induire une lésion de l'épithélium respiratoire. Ces mécanismes de lésion de l'épithélium varient considérablement et regroupent des phénomènes de remaniement de l'épithélium comme la disparition des cellules ciliées jusqu'à la desquamation de l'épithélium respiratoire totale, ou partielle avec maintien des cellules basales ancrées sur la lame basale. De nombreuses molécules, des bactéries ou des virus sont capables d'induire de telles modifications morphologiques de l'épithélium respiratoire. Ces changements incluent des effets ciliostatiques,[1] une hyperplasie mucipare[2] ou une desquamation de l'épithélium de surface.[3,4]

En réponse à une infection bactérienne ou virale, une réponse immunitaire locale peut entraîner la transudation de quantités importantes de protéases sériques vers la lumière du tractus respiratoire, capables à leur tour d'induire des remaniements de l'épithélium respiratoire. L'élastase leucocytaire peut induire une large gamme de remaniements, comme la conversion des cellules muqueuses en cellules séreuses,[5] l'hyperplasie mucipare[6] et la desquamation de l'épithélium de surface.[7] Dans la mucoviscidose, l'infection chronique des voies respiratoires associée à une inflammation chronique, représente des phénomènes majeurs capables d'induire des lésions de l'épithélium respiratoire. Baltimore *et al.*[8] ont montré dans une étude histopathologique, le remaniement intense de l'épithélium respiratoire chez des patients mucoviscidosiques. A la suite d'une lésion de l'épithélium respiratoire, on observe un processus de réparation décrit par Zahm *et al.*[9] à l'aide d'un modèle *in vitro* de lésion-réparation de l'épithélium respiratoire. En fonction de la taille de la lésion, les cellules épithéliales respiratoires répondent par des mécanismes d'étalement, de migration et de prolifération cellulaire[10] afin de restaurer l'intégrité de la barrière épithéliale.[11] La membrane basale mise à nu et en particulier la laminine[12] représentent des sites d'adhérence pour *Pseudomonas aeruginosa*, un des plus importants microorganismes pathogènes isolés dans la mucoviscidose. Les cellules épithéliales respiratoires engagées dans le processus de réparation représentent également des sites majeurs d'attachement pour *P. aeruginosa*. Nous avons décrit récemment l'adhérence préférentielle de *P. aeruginosa* aux cellules épithéliales respiratoires qui s'étalent au bord d'une lésion d'épithélium respiratoire mucoviscidosique ou non-mucoviscidosique. Les modifications phénotypiques des cellules épithéliales respiratoires au cours du mécanisme de réparation, en particulier de celles qui s'étalent au bord de la lésion, sont étroitement liées à une surexpression de récepteurs pour l'adhérence de *P. aeruginosa*. Parmi les molécules capables de servir de récepteurs pour l'adhérence de *P. aeruginosa*, des glycolipides membranaires asialylés comme l'asialo GM1 ont été identifiés à la surface des cellules épithéliales respiratoires participant à la réparation de lésion, et nous avons montré qu'ils jouaient le rôle de récepteurs pour l'adhérence de *P. aeruginosa*.

Alors que l'asialo GM1 représente un site d'adhérence pour *P. aeruginosa* à la surface des cellules épithéliales respiratoires mucoviscidosiques qui réparent une lésion, nous l'avons également retrouvée à la surface de cellules épithéliales respiratoires qui réparent une lésion mais non-mucoviscidosiques.[13] De plus, l'expression apicale d'asialo GM1 diminue au cours du processus de réparation. Ces résultats suggèrent que le défaut primaire de CFTR dans la mucoviscidose ne représente pas l'unique explication de l'adhérence de *P. aeruginosa* aux cellules épithéliales respiratoires.

Pendant le mécanisme de réparation, les cellules épithéliales respiratoires, synthétisent et sécrètent activement de la fibronectine,[14] molécule qui représente également un site d'adhérence pour *P. aeruginosa*.[15] Nous sommes actuellement en train de caractériser le récepteur épithélial pour la fibronectine.

Il semble que les profondes modifications morphologiques des cellules épithéliales respiratoires au cours des mécanismes de lésion et des processus de réparation qui s'en suivent, contribuent de façon majeure aux infections chroniques à *P. aeruginosa* dans la mucoviscidose. Alors que l'affinité de *P. aeruginosa* pour l'épithélium respiratoire qui répare, diminue au cours du processus de réparation en même temps que le nombre de récepteurs asialo GM1,[16] le début du processus de réparation de l'épithélium respiratoire représente une phase critique au cours de laquelle il apparaît nécessaire de protéger l'épithélium respiratoire contre toute nouvelle infection.

#### Références

1. Kanthakumar K, Taylor G, Tsang KWT, *et al.* Mechanisms of action of *Pseudomonas aeruginosa* pyocyanin on human ciliary beat *in vitro. Infect Immun* 1993; **61**: 2848–2853.
2. Huang HT, Haskell A, MacDonald DM. Changes in epithelia secretory cells and potentiation of neurogenic inflammation in the trachea of rats with respiratory tract infections. *Anat Embryol* 1989; **180**: 325–341.
3. Bajolet-Laudinat O, Girod-de Bentzmann S, Tournier JM, *et al.* Cytotoxicity of *Pseudomonas aeruginosa* internal lectin PA I on respiratory epithelial cells in primary culture. *Infect Immun* 1994; **62**: 4481–4487.
4. Plotkowski MC, Puchelle E, Beck G, Jacquot J, Hannoun C. Adherence of type I *Streptococcus pneumonia* to tracheal epithelium of mice infected with influenza A/PRS virus. *Am Rev Respir Dis* 1986; **134**: 1040–1044.
5. Marchand V, Tournier JM, Polette M, Hannion X, Puchelle E. Effect of leucocyte elastase on the expression of antileucoprotease by human adult nasal surface epithelial cells in primary culture. *Eur Resp J* 1995; **8**: **suppl. 19**, 1365.
6. Snider GL, Lucey EC, Christensen PJ, *et al.* Emphysema and bronchial secretory cell metaplasia induced in hamsters by human neutrophil products. *Am Rev Respir Dis* 1984; **129**: 155–160.
7. Plotkowski MC, Beck G, Tournier JM, Bernardo-filtro M, Marques EA, Puchelle E. Adherence of *Pseudomonas aeruginosa* to respiratory epithelium and the effect of leucocyte elastase. *J Med Microbiol* 1989; **30**: 285–293.
8. Baltimore R, Christie C, Walker Smith G. Immunohistopathologic localization of *Pseudomonas aeruginosa* in lung from patients with cystic fibrosis. *Am Rev Respir Dis* 1989; **140**: 1650–1661.
9. Zahm JM, Chevillard M, Puchelle E. Wound repair of human surface respiratory epithelium. *Am J Respir Cell Mol Biol* 1991; **5**: 242–248.
10. Zahm JM, Kaplan H, Doriot F, *et al.* Cell migration and proliferation during the *in vitro* wound repair of the respiratory epithelium. *Micros Res Tech* 1996, in press.
11. Hérard AL, Zahm JM, Pierrot D, Hinnrasky J, Fuchey C, Puchelle E. Epithelial barrier integrity during *in vitro* wound repair of the airway epithelium. Submitted to *Am J Respir Cell Mol Biol*; 1996, in press.
12. Plotkowski MC, Tournier JM, Puchelle E. *Pseudomonas aeruginosa* strains possess specific adhesins for laminin. *Infect Immun* 1996; **64**: 600–605.
13. de Bentzmann S, Roger P, Dupuit F, *et al.* Asialo GM1 as a receptor for *Pseudomonas aeruginosa* adherence to regenerating respiratory epithelial cells. *Infect Immun* 1996, in press.
14. Zahm JM, Hérard AL, Pierrot D, Hinnrasky J, Sheppard D, Puchelle E. Role of fibronectin and its a5b1 integrin receptor during respiratory epithelium wound repair. *Am J Resp Crit Care Med* 1995; **151(4)**: A561.
15. Plotkowski MC, Filho MB, Meirelles MN, Tournier JM, Puchelle E. *Pseudomonas aeruginosa* binds to soluble cellular fibronectin. *Curr Microbiol* 1993; **26**: 91–95.
16. de Bentzmann S, D'Allessandro F, Zahm JM, Pierrot D, Plotkowski MC, Puchelle E. Kinetics of *Pseudomonas aeruginosa* adherence during respiratory epithelial wound repair. *Eur Respir J* 1995; **8(19)**: 42S.

### Expression de CFTR au niveau pulmonaire: quelle est la cellule cible dans la mucovicidose?

*Catherine Figarella et Marc Merten*
Groupe de Recherche sur les Glandes exocrines, Faculté de Médecine, 27 Boulevard Jean Moulin, 13385 Marseille Cedex 5, France

L'atteinte pulmonaire dans la mucoviscidose est caractérisée par une anomalie rhéologique des sécrétions bronchiques accompagnée d'une hyperproduction de mucus induisant par le biais de l'obstruction et d'infections chroniques des bronches une insuffisance pulmonaire progressive léthale. L'hypothèse la plus couramment admise est celle où des défauts de transports ioniques provoqués par la mutation du gène CFTR empêchent l'hydratation du mucus, augmentent sa viscosité et ne permettent plus la clairance des pathogènes inhalés, entraînant ainsi des infections pulmonaires à répétitions. Cependant cette hypothèse permet difficilement d'expliquer la production excessive de mucus et les raisons pour lesquelles les poumons mucoviscidosiques sont colonisés de manière persistante par le *Pseudomonas aeruginosa.* On peut noter par exemple que les malades présentant un défaut de motilité ciliaire entraînant un ralentissement considérable du transport du mucus bronchique ne présentent que des infections légères des voies aériennes.

La compréhension de la physiopathologie pulmonaire dans la mucoviscidose nécessite en premier lieu la caractérisation de l'expression normale de la protéine CFTR dans les différentes cellules de l'arbre respiratoire. Dans une étude princeps, Engelhardt *et al.* ont localisé par des techniques d'hybridation *in situ* et d'immunocytochimie la protéine CFTR et son messager essentiellement au niveau des glandes sous muqueuses et particulièrement dans les cellules séreuses de ces glandes et à une moins grande échelle au niveau des cellules canalaires.[1] L'expression de la protéine est beaucoup plus faible au niveau de l'épithélium de surface. Par la suite Jacquot et coll. ont montré au niveau ultramicroscopique que la protéine était plus spécifiquement associée aux granules secrétoires des cellules séreuses.[2] La protéine est également exprimée à un moindre degré dans une sous-population de cellules épithéliales à tous les niveaux du poumon distal, bronchioles et alvéoles, avec une variation importante du niveau de l'expression suivant les différents échantillons.[3]

Le fait que le siège majoritaire de l'expression de la protéine CFTR soit dans les cellules des glandes sous-muqueuses n'est pas surprenant *a priori* si l'on considère que ces glandes produisent l'essentiel des sécrétions bronchiques dont l'augmentation de viscosité caractérise la maladie. En effet chez l'homme adulte, le volume glandulaire de l'arbre trachéo-bronchique est évalué à environ 4 ml, le volume des cellules caliciformes à mucus de l'épithélium de surface étant le quarantième de cette valeur. Le rôle des glandes dans l'atteinte pulmonaire de la mucoviscidose ne semble, ainsi, ne pas devoir être mis en doute et l'on peut noter que les souris qui à l'état normal ont très peu de glandes sous-muqueuses ne présentent que des atteintes pulmonaires minimes lorqu'elles ont

été complètement déletées du gène CFTR par la technique du knock-out.[4]

La récente mise au point d'un modèle de culture primaire de cellules glandulaires séreuses trachéales humaines nous a permis de montrer que ces cellules, étudiées après leur troisième passage, répondent aux agonistes cholinergiques et adrénergiques et synthétisent les marqueurs des cellules séreuses *in vivo* comme l'inhibiteur sécrétoire ainsi que des glycoconjugués de haut poids moléculaire.[5,6] Nous avons pu ensuite identifier et caractériser l'activité canal chlorure associée à la protéine CFTR dans la membrane apicale de ces cellules en culture.[7] Par ailleurs nous avons montré que les cellules trachéales glandulaires mucoviscidosiques secrétaient de 10 à 50 fois plus de macromolécules que les cellules normales et qu'elles ne répondaient pas aux agents secrétagogues.[8] Ces défauts apparaissent spécifiques des cellules CF car ils n'existent pas dans des cultures de cellules séreuses glandulaires établies à partir de malades atteints d'autres pathologies pulmonaires comme la sarcoidose ou la bronchite chronique obstructive. Ces observations montrent que dans la mucoviscidose, parallèlement à un défaut de transport ionique, il peut y avoir un défaut de sécrétion protéique, suggérant l'idée d'un défaut plus général dans les processus sécrétoires. Enfin, des résultats préliminaires nous ont montré que des cellules glandulaires séreuses normales, cultivées en présence du LPS de *Pseudomonas aeruginosa*, semblaient présenter les mêmes caractéristiques que celles des cellules CF, suggérant que dans la mucoviscidose les cellules se présentent à l'état basal comme des cellules mises dans un contexte inflammatoire.

En conclusion, de nombreux arguments d'ordre génétique, moléculaire et physiologique suggèrent que la cellule trachéale glandulaire séreuse est une des cibles privilégiées de la mucoviscidose. Le fait que les mécanismes de secrétion ionique et macromoléculaire et leurs relations avec la protéine CFTR ne soient pas encore élucidés, ajouté au fait que ces cellules sont profondément enfouies dans la muqueuse, devraient influer sur la recherche des meilleures approches de la thérapie génique de la maladie.

### Références

1. Engelhardt JF, Yankaskas JR, Ernst SA, *et al.* Submucosal glands are the predominant site of CFTR expression in the human bronchus. *Nature Genet* 1992; **2**: 240–247.
2. Jacquot J, Puchelle E, Hinnrasky J, *et al.* Localization of the cystic fibrosis transmembrane conductance regulator in airway secretory glands. *Eur Respir J* 1993; **6**: 169–176.
3. Engelhardt JF, Zepeda M, Cohn JA, Yankaskas JR, Wilson JM. Expression of the Cystic Fibrosis Gene in Adult Human Lung. *J Clin Invest* 1994; **93**: 737–749.
4. Snouwaert JN, Brigman KK, Latour AM, *et al.* An animal model for cystic fibrosis made by gene targeting. *Science* 1992; **257**: 1083–1088.
5. Tournier JM, Merten MD, Meckler Y, Hinnrasky J, Fuchey C, Puchelle E. Culture and characterization of human tracheal gland cells. *Am Rev Respir Dis* 1990; **141**: 1280–1288.
6. Merten MD, Tournier J-M, Meckler Y, Figarella C. Secretory proteins and glycoconjugates synthesized by human tracheal gland cells in culture. *Am J Respir Cell Mol Biol* 1992; **7**: 598–605.
7. Becq F, Merten MD, Voelckel MA, Gola M, Figarella C. Characterization of cAMP dependent CFTR-chloride channels in human tracheal gland cells. *FEBS Letters* 1993; **321**: 73–78.
8. Merten MD, Figarella C. Constitutive hypersecretion and insensitivity to neurotransmitters by cystic fibrosis tracheal gland cells. *Am J Physiol* 1993; **264**: L93–L99.

### Mécanismes du recrutement et de l'activation des neutrophiles durant l'infection pulmonaire à *Pseudomonas aeruginosa*

*Jay A. Nadel*

University of California, San Francisco Cardiovascular Research Institute, San Francisco, CA, USA

Les glandes sous-muqueuses et les cellules caliciformes superficielles des voies aériennes sont normalement responsables des mécanismes protecteurs contre les produits irritants inhalés. Lors des maladies chroniques des voies aériennes, il apparaît une hypersécrétion muqueuse qui est une des causes principales de l'obstruction aérienne qui survient au cours de la mucoviscidose. Un nombre important de neutrophiles et des concentrations élevées de protéases douées d'activité catalytique sont retrouvées dans les expectorations des patients atteints de mucoviscidose, l'élastase des neutrophiles étant le plus puissant des secrétagogues jamais recherché. Ainsi, même après avoir été diluées 30 000 fois, les expectorations des patients atteints de mucoviscidose restent capables de stimuler la sécrétion des cellules glandulaires en culture. Ces résultats indiquent que l'élastase des neutrophiles pourrait être une cause importante de l'hypersécrétion des voies aériennes. Si l'en est ainsi, comment l'élastase est-elle relarguée des neutrophiles?

Dans le cas des cellules caliciformes accessibles à la lumière, les neutrophiles morts sont probablement, au moins en partie, responsables de la sécrétion parce que les expectorations de patients atteints de mucoviscidose contiennent des concentrations élevées d'élastase active en provenance des neutrophiles. Concernant les cellules glandulaires, l'élastase présente dans la lumière ne peut pas être tenue pour responsable de la sécrétion et les neutrophiles intacts ne relarguent pas (ou pas facilement) de l'élastase dans le milieu.

Ces observations suggèrent qu'un processus complexe de signalisation, impliquant la surface des cellules sécrétrices, active des messagers secondaires dans le neutrophile causant l'exocytose de granules contenant de l'élastase au contact de la surface de la cellule sécrétrice. Quel est le mécanisme de recrutement des neutrophiles dans les voies aériennes ? Les expectorations des patients atteints de mucoviscidose contiennent des concentrations élevées d'interleukine 8 (IL-8). Un anticorps dirigé contre l'IL-8 inhibe alors considérablement l'activité chimiotactique des crachats, ce qui suggère que l'IL-8 joue un rôle significatif dans le recrutement des neutrophiles.

Le *Pseudomonas aeruginosa* (PA) est un organisme infectieux important dans la mucoviscidose et les surnageants de PA entraînent une production d'IL-8 au niveau de l'épithélium aérien d'une manière dépendante du temps. L'incubation de voies aériennes normales en provenance de donneurs pour transplantation pulmonaire avec des surnageants de PA entraîne l'expression sélective d'IL-8 au niveau de l'épithélium des voies aériennes. Afin d'examiner si des mécanismes rétroactifs perpétuent ou exagèrent l'expression d'IL-8 et le recrutement des neutrophiles, nous avons réalisé des expériences *in vivo* chez l'animal. Nous avons cloné et exprimé l'IL-8 chez le chien et étudié l'expression de l'IL-8 dans un segment de trachée mis en dérivation. Le surnageant de PA a entraîné une expression sélective et précoce d'IL-8 au niveau de l'épithélium des voies aériennes: ceci était dû à une substance de faible poids moléculaire et soluble dans l'eau produite par le PA. Quelques heures plus tard, l'IL-8 était fortement exprimée dans les neutrophiles recrutés et cela était dû au lipopolysaccharide (LPS) du PA. Ainsi, le recrutement des neutrophiles est potentialisé. Au cours des infections naturelles, le mécanisme devrait continuer jusqu'à ce que les neutrophiles tuent tous les PA, puis s'interrompre rapidement.

En résumé, le recrutement des neutrophiles et le relargage de produits sont des processus compliqués. Les produits de l'épithélium et des neutrophiles constituent un système rétroactif (qui est avantageux pour le sujet normal, mais apparemment pas pour les patients atteints de mucoviscidose). Cette cascade inflammatoire offre de multiples possibilités d'intervention thérapeutique.

### La prévention de la diapédèse leucocytaire associée aux pneumonies bactériennes

*Elaine Tuomanen*
Rockefeller University, New York, USA

On sait de longue date que l'endotoxine est un composé nocif majeur des bactéries Gram négatives. En revanche il n'est pas toujours admis que la paroi cellulaire des bactéries Gram positives ait un effet inflammatoire. Les signes et symptômes d'une pneumonie induite par des parois cellulaires miment ceux observés dans des modèles animaux avec des bactéries vivantes.[1] Les souches cliniques ou leurs dérivés isogéniques de laboratoire, présentent un déficit dans la libération de fragments cellulaires, conduisent à une forme atténuée de la maladie.[2] Ces débris de la paroi cellulaire sont particulièrement bioactifs et certains possèdent une toxicité spécifique envers les cellules épithéliales pulmonaires[3] tandis que d'autres induisent l'afflux de leucocytes ou encore, une perméabilité accrue de l'endothélium pulmonaire.[4] Étant donné que les antibiotiques induisent la libération massive et rapide de l'endotoxine ou des fragments de la paroi cellulaire, il est reconnu que la réduction des lésions au cours d'une infection requiert la diminution de l'état inflammatoire induite par le traitement lui-même. Dans ce contexte, la capacité à moduler la diapédèse leucocytaire, surtout au cours d'un traitement antibiotique d'une infection pulmonaire, est d'un grand intérêt.

Il a été récemment démontré que les médiateurs inflammatoires sont activés par l'endotoxine et les parois cellulaires selon des mécanismes différents.[5] Ceci complique l'élaboration de stratégies visant à moduler de façon négative l'inflammation visant à la réduction des lésions tissulaires. Néanmoins, la diapédèse leucocytaire résulte de la libération de médiateurs au cours des infections par les bactéries Gram positives et négatives. Cet état contribue largement aux dommages pulmonaires. L'inhibition du trafic leucocytaire est alors non seulement une cible raisonnable d'une thérapie accessoire pour une pneumonie grave, mais représente l'une des rares cibles communes aux bactéries Gram positives et négatives.

La migration des leucocytes du sang vers la lumière alvéolaire s'opère en trois étapes. Premièrement, les leucocytes sont marginalisés par l'interaction de glucides présents à leur surface avec des sélectines trouvées sur les cellules endothéliales. Il s'en suit que des sucres circulants ou des analogues de sélectines peuvent rentrer en compétition et inhiber la capacité des leucocytes à atteindre l'endothélium au niveau du site inflammatoire. La seconde étape est l'activation du roulement du leucocyte par des médiateurs tels que le facteur d'activation des plaquettes (PAF) en particulier. Le PAF joue un rôle déterminant dans l'inflammation pulmonaire et des antagonistes du récepteur au PAF s'avèrent tout particulièrement utiles dans ce contexte.[4] La troisième étape consiste en la transmigration proprement dite à partir du sang vers l'alvéole. La famille CD18 des intégrines leucocytaires constitue les molécules clés intervenant dans cette étape. Des anticorps anti-CD18 administrés par voie intraveineuse empêchent l'accumulation de leucocytes au niveau du poumon. De même, des molécules capables de se lier aux récepteurs CD18, telles que les ICAM exprimées à la surface de l'endothélium, empêchent le mouvement des leucocytes. Il existe cependant une exception à ce blocage. Une migration CD18-indépendante unique semblerait rendre compte d'environ 50% des leucocytes pénétrant dans le poumon au cours d'une pneumonie à pneumocoque.[4,6] Étant donné la possibilité théorique d'intervenir à trois étapes de la migration leucocytaire, de nombreuses options pourraient aboutir à une thérapie anti-inflammatoire dirigée contre les leucocytes. Cette approche portera sans doute ses fruits dans le développement de médicaments visant à améliorer le poumon endommagé par une pneumonie ou des états aigus systémiques tel le syndrome de détresse respiratoire aigüe.

### Références

1. Tuomanen E, Rich R, Zak O. Induction of pulmonary inflammation by components of the pneumococcal cell surface. *Am Rev Respir Dis* 1987; **135**: 869–874.
2. Tuomanen E, Pollack H, Parkinson A, *et al.* Microbiological and clinical significance of a new property of defective lysis in clinical strains of pneumococci. *J Infect Dis* 1988; **158**: 36–43.
3. Heiss L, Lancaster J, Corbett J, Goldman W. Epithelial autotoxicity of nitric oxide: role in the respiratory cytopathology of pertussis. *Proc Natl Acad Sci* USA 1994; **91**: 267–270.
4. Cabellos C, MacIntyre DE, Forrest M, Burroughs M, Prasad S, Tuomanen E. Differing roles of platelet-activating factor during inflammation of the lung and subarachnoid space. *J Clin Invest* 1992; **90**: 612–618.
5. Tuomanen E, Austrian R, Masure H. The pathogenesis of pneumococcal infection. *N Engl J Med* 1995; **332**: 1280–1284.
6. Doerschuk CM, Winn RK, Coxson HO, Harlan JM. CD18-dependent and -independent mechanisms of neutrophil emigration in the pulmonary and systemic microcirculation of rabbits. *J Immunol* 1990; **144**: 2327–2333.

### Les gènes des sérine protéinases des leucocytes

*R. A. Stockley et K. McConn*
Department of Medicine, Queen Elizabeth Hospital, Edgbaston, Birmingham, UK

Les leucocytes contiennent plusieurs sérine protéinases dont l'élastase des neutrophiles, la protéinase 3 (autoantigène de la maladie de Wegener) et la cathepsine G. Ces trois enzymes sont impliquées dans la pathogénèse des maladies pulmonaires chroniques, l'élastase et la protéinase 3 ayant la capacité de provoquer un emphysème pulmonaire expérimental. De plus, il a été mis en évidence que l'élastase du neutrophile et la cathepsine G pouvaient jouer un rôle dans la physiopathologie de plusieurs maladies pulmonaires chroniques.

Peu d'informations concernant ces protéinases et leur régulation sont disponibles. Les gènes codant pour ces protéinases sont localisés sur différents chromosomes, le gène de l'élastase du neutrophile et celui de la protéinase 3 sont localisés sur le chromosome 19, D13.3[1] alors que celui de la cathepsine G est localisé sur le chromosome 14 Q 11.2.[2] Ces gènes sont exprimés au cours de la différenciation du neutrophile dans la moëlle osseuse et actuellement aucun argument ne permet de dire si ces gènes sont exprimés dans une cellule mature, complètement différenciée. Le gène le mieux caractérisé à l'heure actuelle est celui de l'élastase; il est exprimé au stade promyélocyte lors de la différenciation du neutrophile et son expression est donc spécifique d'un tissu et d'une étape du processus de différenciation.

La région 5′ flanquante du gène de l'élastase contient une CAAT box, une TATA box et des séquences riches en CG, éléments en faveur de la présence d'un promoteur fonctionnel. Des expériences de délétion de nucléotides ont suggéré que d'autres séquences régulatrices étaient présentes dans la région située à 200 paires de bases en amont du codon d'initiation. Han et collaborateurs[3] ont démontré la présence de séquences régulatrices soit positives (–149 à –102 paires de bases) ou négatives (–196 à –153) alors que Srikanth et Rado[4] ont identifié une autre séquence de régulation positive située de –106 à –76 paires de bases. Ces séquences ne sont actives que dans les cellules au stade promyélocytique, et pas dans d'autres lignées cellulaires telles la lignée Hep G2 ou HELA, mettant en evidence la spécificité cellulaire de ce type de régulation.

Des études réalisées sur des lignées promonocytiques et promyélocytiques ont révélé que l'expression du gène de l'élastase peut être diminuée par des facteurs de différenciation. De plus, il a été montré que l'addition d'oligonucléotides anti-sens des transcrits du gène de la protéinase 3 dans la lignée HL-60 induisait la différenciation vers la lignée monocytaire.[5] La signification de ce phénomène reste encore mal connue.

L'importance physiopathologique de l'élastase a été mise en évidence dans des situations de déficit génétique spécifiques. Chez l'animal, la souris beige représente un variant génétique caractérisé par une absence d'expression du gène de l'élastase. Les neutrophiles de ces animaux sont dépourvus d'élastase. Au cours d'infections expérimentales, il a été montré que les neutrophiles étaient recrutés normalement et, fait important, la survie de ces animaux était augmentée.[6] Ces résultats ont donc mis l'accent sur

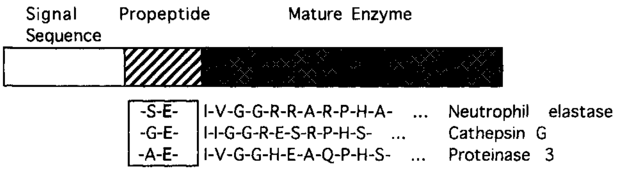

FIG. 1. Représentation schématique des transcrits des gènes des protéinases démontrant le peptide signal et le dipeptide pro-enzyme. La séquence de l'extrémité N-terminale de la pro-enzyme est détaillée.

l'état d'inflammation associé à l'infection, démontrant ainsi que l'élastase du neutrophile pouvait avoir des effets délétères, et ont confirmé l'importance de cette sérine protéinase dans les infections chroniques chez l'homme.[7]

Chez l'homme, l'équivalent du déficit observé chez la souris beige est le syndrome de Chediak Higashi, au cours duquel les neutrophiles matures présentent une anomalie dans la formation des granules. Ces neutrophiles ont un défaut du chimiotactisme et ne contiennent pas d'élastase. Des études ont montré que la cathepsine G est aussi absente de ces neutrophiles matures, et des résultats très récents permettent d'affirmer que ces neutrophiles sont aussi déficients en protéinase 3 (observations non publiées obtenues en collaboration avec P. Hiemstra, Leiden). Cependant, des études des cellules de la moëlle osseuse ont montré que les neutrophiles immatures expriment les gènes de l'élastase et la cathepsine G et que ces enzymes sont présentes avant la maturation complète.[8] Ces sérine protéinases étant codées par des gènes localisés à des endroits différents dans le génome et deux d'entre elles au moins étant exprimées à des stades précoces de la différenciation dans la moëlle osseuse, il semblerait logique de penser que le déficit du syndrome de Chediak Higashi est un déficit commun touchant probablement des modifications post-traductionnelles. Il a été montré que les trois sérine protéinases étaient produites sous forme de pré-pro-enzyme. La séquence signal de la partie amino terminale est clivée pour donner la pro-enzyme, puis un dipeptide est clivé à un site précis comme le montre la figure. Des études récentes ont suggéré que l'enzyme déficiente pourrait être une cystéine dipeptidase mais cette hypothèse demande à être confirmée.

L'étude des maladies pulmonaires a permis de mettre en évidence une quantité accrue d'élastase dans les neutrophiles de patients souffrant de bronchiectasie par rapport à des témoins du même âge ou à des patients souffrant d'un emphysème.[9] Cette augmentation de l'expression de l'enzyme n'est pas unique car les neutrophiles de patients ayant une arthrite rhumatoide ou de patients diabétiques contiennent une quantité plus importante d'élastase. Les mécanismes qui soutendent cette production accrue d'enzyme ne sont pas connus mais apparaissent influencer les mécanismes de destruction des tissus. Ces résultats suggèreraient qu'il existe des mécanismes de modulation de la quantité d'élastase du neutrophile et que ceci se produirait probablement au cours de la différenciation. L'augmentation du contenu en élastase peut résulter d'une augmentation de la transcription, d'une traduction de l'ARN messager plus efficace, de modifications dans la maturation intracellulaire, le stockage ou le transport à la membrane cellulaire. Des études approfondies de ces processus devraient permettre de déterminer le mécanisme exact et d'apporter également des informations sur des cibles thérapeutiques potentielles pour la modulation de l'expression de ces protéinases.

### Références

1. Zimmer M, Medcalf RL, Fink TM, Mattmann C, Lichter P, Jenne DE. Three human elastase-like genes coordinately expressed in the myelomonocyte lineage are organized as a single genetic locus on 19 pter. *Proc Natl Acad Sci USA* 1992; **89**: 8215–8219.
2. Heusel JW, Hanson, RD, Silverman GA, Ley TJ. Structure and expression of a cluster of human haematopoietic serine protease genes found on chromosome 14q 11.2. *J Biol Chem* 1991; **266**: 6152–6158.
3. Han J, Unlap, T, Radion TA. Expression of the human neutrophil elastase gene: positive and negative transcriptional elements in the 5′ flanking region. *Biochem Biophys Res Comm* 1991; **181**: 1462–1468.
4. Srikanth S, Rado TA. A 30-base pair element is responsible for the myeloid-specific activity of the human neutrophil elastase promoter. *J Biol Chem* 1994; **269**: 32626–32633.
5. Bories, D, Raynal M, Solomon DH, Darzynklewicz Z, Cayre YE. Down-regulation of serine proteinase myeloblastin causes growth arrest and differentiation of promyelocytic leukaemia cells. *Cell* 1989; **50**: 956–968.
6. Tanaka E, Yuba Y, Sato A, Kuze, F. Effects of the beige mutation on respiratory tract infection with *Pseudomonas aeruginosa* in mice. *Exp Lung Res* 1994; **20**: 351–366.
7. Stockley RA. Bronchiectasis – new therapeutic approaches based on pathogenesis. In: *Clinics in Chest Medecine*, Vol. 8, No. 3. Philadelphia: W. B. Saunders, 1987: 481–494.
8. Burnett D, Ward, CJ, Stockley RA, *et al.* Neutrophil elastase and Cathepsin G protein in messeger RNA expression in bone marrow from a patient with Chediak higashi syndrome. *J Clin Path* 1995; **48**: M28–M34.
9. Burnett D, Chamba A, Hill SL, Stochley RA. Neutrophils from subjects with chronic obstructive lung disease show enhanced chemotaxis and extracellular proteolysis. *Lancet* 1987; **11**: 1043–1046.

### Utilisation d'inhibiteurs de protéinases en thérapie

*Gerd Döring* et Gabriel Bellon***

*Hygiene Institut, Universität Tübingen, Germany and

** Hôpital Lyon-Sud, Lyon, France

De nombreux travaux ont maintenant bien démontré que les conséquences de l'activation des neutrophiles dans les voies aériennes des patients atteints de mucoviscidose sont en partie responsables des problèmes respiratoires et de la mortalité observée dans la mucoviscidose. Lors de leur activation, les neutrophiles libèrent des métalloprotéinases lysosomiales et des sérine protéinases; outre la collagénase active et la cathepsine G, des quantités importantes d'élastase ont été détectées dans les lavages broncho-alvéolaires ou dans les expectorations des patients atteints de mucoviscidose.[1,2] Apparemment, un déséquilibre dans la balance élastase/anti-élastase est présent dans les voies aériennes inflammées au cours de la mucoviscidose et une forte proportion de l'inhibiteur majeur de l'élastase, l'alpha1-inhibiteur de protéase (alpha1-PI) est digérée par protéolyse et inactivée.[1] Les autres inhibiteurs de protéases, l'inhibiteur des leucoprotéases sécrétoires (secretory leukocyte protéase inhibitor ou SLPI) et l'alpha2-macroglobuline apparaissent jouer un rôle mineur dans la protection contre l'élastase, de l'arbre respiratoire inférieur.

L'élastase du neutrophile joue un rôle majeur dans la physiologie de l'inflammation chronique dans la mucoviscidose ainsi que dans d'autres maladies. Outre l'inactivation de l'alpha1-inhibiteur de protéase, l'élastase peut dégrader l'élastine pulmonaire et la fibronectine, stimuler la sécrétion des glandes des voies aériennes, réduire la fréquence des battements ciliaires et entraver l'opsonisation et la phagocytose au niveau des immunoglobulines, du complément et de son récepteur CR1 sur le neutrophile.[1] Ces évènements pourraient commencer très précocément dans la vie du patient atteint de mucoviscidose car des taux très élevés d'élastase ont été trouvés chez des patients agés de 6 mois au plus.[3,4] Chez des patients plus agés, de forts taux d'élastase peuvent être présents non seulement chez des patients atteints très sévèrement mais aussi chez des patients stables cliniquement avec une atteinte pulmonaire légère.[5]

Ce déséquilibre de la balance élastase/anti-élastase suggère une stratégie visant à augmenter le niveau d'antiprotéase dans le poumon par l'administration d'inhibiteurs appropriés.[1] Parmi les nombreux inhibiteurs développés pour l'élastase, certains d'entre eux pourraient être utilisés chez l'homme, l'alpha1-inhibiteur de protéase est l'un d'entre eux. Un essai mené sur un nombre réduit de patients atteints de mucoviscidose et utilisant un aérosol de l'alpha1-inhibiteur de protéase pendant plusieurs semaines a donné des résultats encourageants;[7] Lorsque 12 patients reçoivent un aérosol de 1,5–3,0 mg/kg toutes les 12 heures et pendant une semaine, l'activité de l'élastase était supprimée dans le fluide péri-épithélial et la capacité anti-élastase du neutrophile était restaurée lorsque la concentration de alpha1-inhibiteur de protéase avait atteint 8 μM/l. De plus, le pouvoir microbicide *in vitro*, envers *Pseudomonas aeruginosa* était augmenté. Cependant, dans un autre essai mené à Lyon, au cours duquel la même dose d'alpha1-inhibiteur de protéase était administrée deux fois par jour pendant trois jours, ces résultats ne furent pas confirmés. Lorsque l'activité de l'élastase était mesurée chaque jour dans les expectorations et dans les lavages broncho-alvéolaires, un jour avant et 8 jours après aérosolisation, aucune réduction significative de l'activité de l'enzyme n'a été trouvée. Des résultats positifs ont été obtenus dans une étude multicentrique d'aérosol d'alpha1-inhibiteur de protéase comprenant 22 patients atteints de mucoviscidose et au cours de laquelle 100 mg, 200 mg ou 350 mg étaient administrés toutes les 12 heures pendant 4 semaines. Chez tous les patients à l'exception d'un seul, l'activité de l'élastase a été diminuée et/ou sa capacité à inhiber l'élastase exogène a été augmentée dans le fluide péri-épithélial. Cependant l'activité de l'élastase de l'expectoration n'était pas corrélée avec l'activité du fluide épithélial. En conclusion, des niveaux d'alpha1-inhibiteur de protéase effectivement et biochimiquement active (prolastin) peuvent être administrés sans risque mais d'autres études sont nécessaires afin de procéder à une meilleure évaluation de l'application de l'alpha1-inhibiteur de protéase dans la mucoviscidose.

### Références

1. Döring G, Knight R, Bellon G. Immunology of cystic fibrosis. In: Hodson ME, Geddes DM, eds. *Cystic Fibrosis*. London: Chapman and Hall, 1994; 99–129.
2. Goldstein W, Döring G. Lysosomal enzymes and proteinase inhibitors in the sputum of patients with cystic fibrosis. *Am Rev Respir Dis* 1986; **134**: 49–56.
3. Khan TZ, Wagener JS, Bost T, Martinez J, Accurso FJ, Riches DWH. Early pulmonary inflammation in infants with cystic fibrosis. *Am J Crit Care Med* 1995; **151**: 1075–1082.
4. Birrer P, McElvaney NG, Rüdeberg A *et al.* Protease-antiprotease imbalance in the lungs of children with cystic fibrosis. *Am J Crit Care Med* 1994; **150**: 207–213.
5. Konstan MW, Hilliard KA, Norvell TM, Berger M. Bronchoalveolar lavage findings in cystic fibrosis patients with stable, clinically mild lung disease suggest ongoing infection and inflammation. *Am J Crit Care Med* 1994; **150**: 448–454.
6. McElvaney NG, Hubbard RC, Birrer P, *et al.* Aerosol α1-antitrypsin treatment for cystic fibrosis. *Lancet* 1991; **337**: 392–394.
7. Berger M, Konstan MW, Hilliard J. Aerosolized prolastin (α1-protease inhibitor) in CF. Abstr. presented at the *9th Annual North American CF Conference*, Dallas October 1995.

### L'activité oxydative du polynucléaire neutrophile

*Véronique Witko-Sarsat et Béatrice Descamps-Latscha*

INSERM U25, Hôpital Necker 161, rue se Sèvres, 75015 Paris, France

Afin d'assurer leur fonction principale qui est la défense de l'hôte contre les agents pathogènes, les polynucléaires neutrophiles disposent de deux types de mécanismes microbicides.[1]

Les systèmes indépendants de l'oxygène dont les molécules effectrices sont les protéases et les protéines antibiotiques préformées et stockées dans les granules azurophiles. Parmi celles-ci, l'élastase, sérine protéinase libérée lors de l'activation cellulaire, a été particulièrement bien étudiée dans le contexte de la mucoviscidose. Présente en grande quantité dans les voies aérienne des patients, elle témoigne ainsi de l'importance des médiateurs dérivés du polynucléaire dans les processus inflammatoires caractéristiques de cette maladie.

Le deuxième type de mécanisme microbicide du polynucléaire neutrophile correspond aux systèmes dépendants de l'oxygène aboutissant à la production d'oxydants.[2] Contrairement aux protéases, ils n'ont pas fait l'objet d'études extensives dans le contexte de la mucoviscidose. Il a cependant été montré, qu'au cours de la mucoviscidose, la balance oxydant/antioxydant était déséquilibrée et qu'il y aurait donc un stress oxydant.

Les systèmes microbicides dépendants de l'oxygène mettent en jeu la capacité d'activation métabolique oxydative du polynucléaire neutrophile ou explosion respiratoire qui fait suite à l'activation de la NADPH oxydase.[3] Ce complexe est une chaîne de transport d'électrons formée de sous-unités membranaires dont un cytochrome β membranaire de faible potentiel constitué lui-même d'une sous unité alpha ($p^{21phox}$ pour phagocyte oxydase) et d'une sous unité béta ($gp91^{phox}$) fortement glycosylée et comportant un site de fixation du NADPH et un autre pour le FAD. Les sous-unités cytoplasmiques sont $p47^{phox}$ (qui se lie à gp91 après phosphorylation par la protéine kinase C), $p67^{phox}$ et une protéine de liaison de GTP, Rac-1 ou Rac-2, qui hydrolyse GTP en GDP et favorise ainsi la dissociation du complexe actif.

Après assemblage, le complexe multimoléculaire de la NADPH-oxydase est fonctionnel, la NADPH-oxydase peut alors catalyser la réduction univalente de l'oxygène en anion superoxyde aux dépens du NADPH aboutissant à la formation d'anion superoxyde ($O_2^{-}\cdot$). La dismutation de l'anion superoxyde spontanée ou induite par la superoxyde dismutase (SOD) aboutit à la formation de peroxyde d'hydrogène ($H_2O_2$) puis, en présence de fer (réaction de Fenton) à la production de radicaux hydroxyle OH (voir Fig. 1).

Ensuite intervient la myéloperoxydase, seconde enzyme importante des mécanismes dépendants de l'oxygène. La myéloperoxydase est une enzyme stockée dans les granules azurophiles et représente 5% de son poids sec; elle catalyse la formation d'acide hypochloreux (HOCl) à partir de $H_2O_2$ et de chlorures présents dans le cyto-

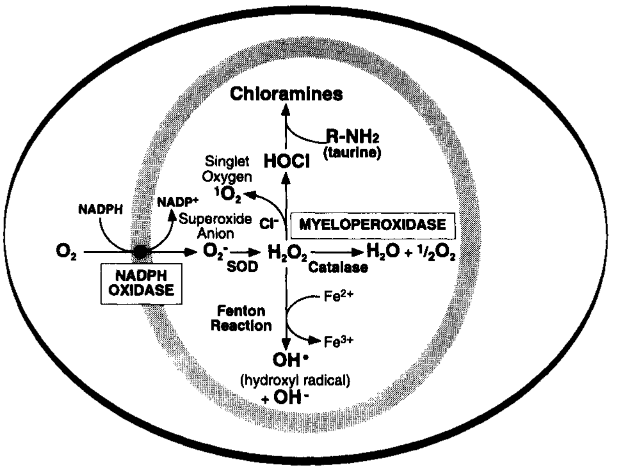

FIG. 1. Réactions de formation des oxydants suite à l'activation du polynucléaire neutrophile.

plasme.[4] La formation d'oxygène singulet, autre espèce microbicide a également été décrite.[5] L'acide hypochloreux peut réagir avec des amines endogènes ($R\text{-}NH_2$) pour former des chloramines (RNH-Cl).[6] La majorité des chloramines seraient formées à partir de la taurine, acide béta-aminé très abondant dans les leucocytes et considérée comme un 'piégeur' sélectif d'HOCl. En effet, les métabolites dérivés de l'oxygène tels l'anion superoxyde, $H_2O_2$ ou HOCl sont des espèces particulièrement réactives et labiles alors que les chloramines sont assez stables pour s'accumuler dans le milieu extracellulaire et ont, de ce fait été baptisées 'oxydants à vie longue'. La présence d'oxydants à vie longue a récemment été rapportée dans les expectorations de sujets atteints de mucoviscidose.

D'après les études *in vitro*, l'anion superoxyde et $H_2O_2$, premiers métabolites générés lors de l'activation de NADPH-oxydase ne semblent pas être les espèces chimiques les plus efficaces dans la destruction des microorganismes. En fait la puissance des mécanismes microbicides réside dans la formation d'HOCl, espèce la plus toxique et la plus réactive formée par les phagocytes,[8] ou peut-être via la formation d'oxygène singulet.[5]

L'exploration de l'activation métabolique oxydative du polynucléaire neutrophile des sujets atteints de mucoviscidose a donné des résultats discordants, en raison, de la grande hétérogénéité, d'une part dans l'état clinique des malades testés, et, d'autre part, dans les protocoles de stimulation et les méthodes utilisées.

Il semblerait que le défaut de transduction intracellulaire présent dans la cellule épithéliale ne soit pas retrouvé au niveau du polynucléaire neutrophile. En effet, l'élévation d'AMP cyclique intracellulaire par différents agents pharmacologiques (adrénaline, théophylline et forskoline) a le même effet inhibiteur sur des fonctions cellulaires du polynucléaire neutrophile telles la production d'anion superoxyde, la dégranulation, la dépolarisation membranaire chez le sujet atteint de mucoviscidose que chez le sujet normal.[9] Il a été montré que la chimioluminescence amplifiée par le luminol d'une préparation de polynucléaires neutrophiles stimulés par le zymosan a une intensité identique chez les sujets atteints de mucoviscidose que chez les témoins. Cependant, la cinétique du phénomène est différente: le temps mis pour atteindre la valeur maximale de chimioluminescence (ou pic) est inférieur chez les patients atteints de mucoviscidose; de plus, ce paramètre est corrélé à l'état clinique des malades. Ces auteurs concluent donc à une hyperréactivité des polynucléaires neutrophiles, qui apparaissent pré-activés.[10] Enfin, d'autres travaux portant sur les monocytes et les polynucléaires neutrophiles de malades atteints de mucoviscidose ont montré une augmentation de chimioluminescence amplifiée par le luminol après stimulation par le f-MLP, le PMA et le A23187.[11]

Il est en effet difficile, dans le cas de la mucoviscidose de faire la part entre l'activation ou priming des polynucléaires faisant suite aux infections et une éventuelle anomalie de type plus constitutif du polynucléaire neutrophile. L'étude des neutrophiles de sujets hétérozygotes pour la mutation du CFTR, qui sont totalement asymptomatiques cliniquement, pourrait fournir une bonne alternative et permettre de discriminer entre ces deux possibilités. Il a été rapporté que les monocytes de sujets homozygotes et hétérozygotes avaient une capacité accrue d'adhérer sur le verre.[12] Dans une récente étude menée dans notre laboratoire, nous avons mis en évidence une anomalie de la formation des oxydants chlorés dépendants de la myéloperoxydase chez les enfants mais aussi chez les parents par rapport aux sujets témoins. Le mécanisme pourrait impliquer l'échangeur sodium/proton car cette hyperactivité des polynucléaires neutrophiles est corrigée par des agents bloqueurs de l'échangeur tels l'amiloride ou l'EIPA, ou dans un milieu dépourvu de sodium (Witko-Sarsat *et al.*, soumis pour publication).

En conclusion, le polynucléaire neutrophile demeure la cellule au coeur du processus inflammatoire, la plus spécialisée et la plus efficace dans la génération d'oxydants. Il apparait donc important d'explorer les mécanismes de formation, d'étudier les conséquences de cette libération massive d'oxydants ainsi que leur importance *in vivo*.

### Références

1. Witko-Sarsat V, Descamps-Latscha B. Phagocyte-derived oxidants and proteinases as immunomodulatory mediators in inflammation. *Mediat Inflamm* 1994; **3**: 257–273.
2. Babior BM. Oxygen-dependent microbial killing by phagocytes. *N Engl J Med* 1978; **298**: 659–668.
3. Gallin JI, Leto TL, Rotrosen D, Kwong CH, Malech HL. Delineation of the phagocyte NADPH oxidase through studies of chronic granulomatous diseases of childhood. *Curr Opin Immunol* 1991; **4**: 53–56.
4. Klebanoff SJ. Myeloperoxidase-halide-hydrogen peroxide antibacterial system. *J Bacteriol* 1969; **95**: 2131–3138.
5. Allen RL, Stjernholm R, Steele RH. Evidence for the generation of an electronic excitation state(s) in human polymorphonuclear leukocytes and its participation in bactericidal activity. *Biochem Biophys Res Commun* 1972; **47**: 679–684.
6. Weiss SJ, Lampert MB, Test ST. Long-lived oxidants generated by human neutrophils: characterization and bioactivity. *Science* 1983; **222**: 623–628.
7. Witko-Sarsat V, Delacourt C, Rabier D, Nguyen AT, Descamps-Latscha B. Presence of long-lived oxidants in sputum of cystic fibrosis patients. *Am J Resp Crit Care Med* 1995; **152**: 1910–1912.
8. Passo SA, Weiss SJ. Oxidative mechanisms utilized by human neutrophils to destroy *Escherichia coli*. *Blood* 1984; **3**: 1361–1368.
9. Suter SI, Chevallier KH, Krause Lew DP. Effect of cyclic adenosine monophosphate elevations on functional responses of polymorphonuclear leukocytes from patients with cystic fibrosis. *Pediatr Pulmonol* 1989; **6**: 237–241.
10. Roberts RL, Stiehm ER. Increased phagocytic cell chemiluminescence in patients with cystic fibrosis. *Am J Dis Child* 1989; **143**: 44–950.
11. Kemp T, Schram-Doumont A, Van Geffel R, Kram R, Szpirer C. Alteration of the N-formyl-methionyl-leucyl-phenylalanine-induced response in cystic fibrosis neutrophils. *Pediatr Res* 1986; **20**: 520–526.
12. Regelmann WE, Lunde LM, Porter PT, Quie PG. Increased monocyte chemiluminescence in cystic fibrosis patients and in their parents. *Pediatr Res* 1986; **20**: 619–622.

### Le rôle du stress oxydant dans la mucoviscidose

*Frank J. Kelly*
Cardiovascular Research, The Rayne Institute,
St Thomas' Hospital, London, UK

Bien que l'anomalie génétique responsable de la mucoviscidose soit bien caractérisée, les évènements conduisant à la destruction progressive du tissu pulmonaire et la diminution consécutive de la fonction respiratoire, restent mal connus. C'est pourquoi notre groupe a étudié depuis quelques années le rôle spécifique du stress oxydant et des défenses anti-oxydantes dans la physiopathologie de la mucoviscidose.[1–5] Deux faits importants sont apparus dans cette série d'études. Premièrement, aucun déficit important d'anti-oxydants liposolubles tels que l'$\alpha$-tocophérol (vitamin E) n'a été retrouvé. Ceci reflète probablement le bon état clinique et nutritionnel des sujets atteints de mucoviscidose et suivis dans les établissements spécialisés. Deuxièmement, plus surprenant, malgré des concentrations en substances anti-oxydantes relativement normales, les sujets atteints de mucoviscidose montrent des signes en faveur d'un stress oxydant important.

Le plasma des sujets atteints de mucoviscidose a une capacité de piéger les radicaux de l'oxygène diminuée par rapport au plasma de sujets normaux, suggérant une susceptibilité accrue au stress oxydatif.[1] La présence de lipides oxydés

(hydroperoxydes) et de protéines oxydées (carbonyles) dans le plasma de sujets atteints de mucoviscidose, suggère que les lésions tissulaires sont dues aux radicaux libres.[2] De plus, nous avons montré que le produit d'oxydation de l'ADN, 8-hydroxydeoxyguanosine est présent dans les urines de sujets atteints de mucoviscidose.[3] Comme nous le mentionnons ci-dessus, fait intéressant mais surprenant, le stress oxydatif est décelable chez des enfants atteints de mucoviscidose dont les défenses anti-oxydantes (vitamine E, acide ascorbique, acide urique et sulphydryles totaux plasmatiques) sont normales. Ces résultats confirment donc que la production accrue de radicaux libres est une des composantes de la mucoviscidose, et que les concentrations d'antioxydants circulants sont insuffisantes chez de nombreux patients atteints de mucoviscidose pour pallier les conséquences de l'augmentation des oxydants.

*Stress oxydant et déclin de la fonction respiratoire dans la mucoviscidose*

La mise en évidence de marqueurs de stress oxydant dans le plasma ou les urines a permis de donner une base biochimique aux lésions des radicaux libres dans la mucoviscidose, mais l'importance clinique de ces résultats reste à déterminer. Nous avons récemment trouvé que la diminution liée à l'âge de la fonction respiratoire chez les enfants atteints de mucoviscidose est associée à une diminution de l'alpha-tocophérol, l'acide ascorbique, et des concentrations de sulphydryle. De plus, la réduction de la fonction respiratoire liée à l'âge est corrélée avec une élévation des taux plasmatiques de malondialdehyde chez les enfants atteints de mucoviscidose.[4] Enfin, les sujets avec une diminution sévère de la fonction respiratoire (VEMS < 50% normale) ont des concentrations plasmatiques en hydroperoxydes lipidiques plus élevées que celles des sujets ayant un déficit de la fonction respiratoire mineur ou modéré (VEMS > 50% normale). Ces résultats nous incitent à rechercher l'existence d'une relation entre l'état de la fonction respiratoire et l'intensité du stress oxydant.

*Défenses anti-oxydantes et marqueurs du stress oxydant dans les expectorations*

Les expectorations viennent des voies aériennes, et représentent donc une source de renseignements plus intéressante que le plasma pour étudier les marqueurs du stress oxydant et les anti-oxydants. Dans une étude pilote conduite chez 11 sujets atteints de mucoviscidose, nous avons démontré qu'il est possible, avec des modifications mineures des méthodes existantes, de mesurer l'alpha-tocophérol, l'acide urique, l'acide ascorbique et les sulphydryles totaux dans les expectorations. Les concentrations et la composition de ces anti-oxydants montre que les expectorations sont un substitut raisonnable de ce qui se passe dans les voies aériennes supérieures. Malgré la présence de nombreux anti-oxydants extracellulaires, des stigmates d'une oxydation lipidique et protéique sont observés dans les expectorations des sujets atteints de mucoviscidose. Nous n'avons pas eu encore la possibilité de déterminer si les marqueurs du stress oxydant dans les expectorations sont corrélés avec ceux présents dans le plasma des mêmes patients. De même, l'existence éventuelle d'une relation entre les défenses anti-oxydantes et les marqueurs de stress oxydant présents dans les expectorations, et la fonction respiratoire mériterait d'être explorée.

*Thérapeutique anti-oxydante chez les sujets atteints de mucoviscidose*

Deux résultats de notre équipe suggèrent qu'une supplémentation en anti-oxydants, au dessus des taux normaux, pourrait apporter un bénéfice thérapeutique aux sujets atteints de mucoviscidose. Premièrement, les marqueurs de stress oxydant, qui ne sont pas présents chez les enfants sains de même âge, sont présents chez beaucoup de sujets atteints de mucoviscidose, dont les défenses anti-oxydantes circulantes sont par ailleurs normales pour l'âge. Deuxièmement, les sujets atteints de mucoviscidose, ayant des concentrations plasmatiques d'anti-oxydants les plus élevées, ont la meilleure fonction respiratoire. Nous avons émis l'hypothèse que les sujets atteints de mucoviscidose subissent des périodes cycliques de stress oxydant associées avec des infections respiratoires chroniques et que le stress oxydant, lorqu'il est supérieur aux défenses anti-oxydantes pulmonaires, entraine des lésions pulmonaires.[5] L'hypothèse que ce stress oxydant combiné au déséquilibre protéase/antiprotéase, contribue à la pathologie pulmonaire de la mucoviscidose et à l'altération de la fonction respiratoire est hautement probable. Combattre le stress oxydant avec une thérapeutique anti-oxydante pourrait aider à diminuer les lésions pulmonaires, et ainsi retarder l'altération de la fonction respiratoire.

### Références

1. Langley SC, Brown RK, Kelly FJ. Reduced free radical-trapping capacity and altered plasma antioxidant status in cystic fibrosis. *Pediat Res* 1993; **33**: 247–250.
2. Brown RK, Kelly FJ. Evidence for increased oxidative damage in patients with cystic fibrosis. *Pediat Res* 1994; **36**: 487–493.
3. Brown RK, McBurney A, Lunec J, Kelly FJ. Oxidative damage to DNA in patients with cystic fibrosis *Free Rad Biol Med* 1995; **8**: 801–806.
4. Brown RK, Wyatt H, Price J, Kelly FJ. Increased oxidative damage and decreased pulmonary function in cystic fibrosis. *Eur Res J* 1996; **9**: 334–339.
5. Brown RK, Kelly FJ. Role of free radicals in the pathogenesis of cystic fibrosis. *Thorax* 1994; **49**: 738–742.

### Les stratégies thérapeutiques contre les lésions cellulaires induites par les radicaux de l'oxygène?

*Dieter Worlitzsch, Gunda Herberth et Gerd Döring*

Hygiene Institut, Universität Tübingen, Germany

Dans la mucoviscidose, la maladie autosomale récessive la plus fréquente dans les populations blanches, les sécrétions anormales des glandes exocrines conduisent à des infections bronchiques chroniques avec des agents pathogènes bactériens opportunistes tels que le *Pseudomonas aeruginosa*. Ceci entraîne le recrutement d'un grand nombre de polynucléaires neutrophiles (PN) dans les voies aériennes infectées, dont l'activation aboutit à la libération d'enzyme lysosomiaux[1] ainsi qu'une augmentation de leur consommation d'oxygène et la production de métabolites dérivés de l'oxygène tels que l'anion superoxyde, le peroxyde d'hydrogène, les radicaux hydroxyles et peut-être l'oxygène singulet. Comme pour les protéinases lysosomales, ces dérivés réduits de l'oxygène sont libérés non seulement dans le phagolysosome mais aussi hors du phagocyte où ils peuvent exercer des effets délétères sur les tissus avoisinants.

Il n'est pas encore certain que les dérivés réduits de l'oxygène produits par les PN stimulés contribuent aux lésions pulmonaires au cours de la mucoviscidose, comme cela a déjà été suggéré dans d'autres maladies des voies aériennes.[2] Cependant, un certain nombre de faits sont en faveur de cette hypothèse; ainsi, les concentrations élevées dans les crachats de myéloperoxydase (MPO), une enzyme dérivée des PN qui transforme le peroxyde d'hydrogène ($H_2O_2$) en dérivés chlorés hautement réactifs, ont été détectés chez les sujets atteints de mucoviscidose[3–6] et une corrélation inverse entre la fonction respiratoire et les concentrations de MPO a été observée.[4–6] En outre, un déficit en glutathion[7] et une augmentation de la peroxydation lipidique[8] ont également été décrits chez les sujets atteints de mucoviscidose. La présence de piégeurs endogènes tels que la superoxyde dismutase (SOD) qui dismute l'anion superoxyde en peroxyde d'hydrogène, et la catalase qui transforme le peroxyde d'hydrogène en eau et oxygène, n'a pas encore été étudiée dans les crachats ou le liquide de lavage alvéolaire. Le gène de la catalase humaine dans les cellules épithéliales des voies aériennes est un gène de type 'domestique' qui ne peut être augmenté par l'hyperoxie.[9]

L'inactivation protéique secondaire à une attaque oxydative n'a pas encore été clairement démontrée dans la mucovisidose et il serait particulièrement intéressant d'étudier une éventuelle inactivation de l'alpha1-antitrypsine. De nombreuses études ont montré que la Met[358], un résidu important du site de liaison de la sérine protéinase, est particulièrement sensible à l'attaque oxydative, et que ceci entraîne une réduction importante de sa capacité de liaison avec l'élastase des neutrophiles.[10]

Si l'on admet l'existence d'un déséquilibre oxydants/anti-oxydants au cours de la mucoviscidose, quels moyens thérapeutiques permettront de protéger l'épithélium pulmonaire, les protéines essentielles, les acides gras, les carbohydrates et l'ADN des lésions induites par le stress oxydant? Certaines stratégies sont actuellement développées, incluant rhSOD[11] ou le sélénium,[12] l'aérosolisation de N-acétylcystéine comme précurseur du glutathion[13] et l'augmentation de la barrière anti-oxydante à la surface de l'épithélium de sujets atteints de mucoviscidose avec des vecteurs adénoviraux recombinants déficients pour la réplication et contenant l'ADNc de la catalase humaine,[2] ou de la catalase associée à des liposomes.[14]

### Références

1. Döring G, Knight R, Bellon G. Immunology of cystic fibrosis. In: Hodson ME, Geddes MD, eds. *Cystic Fibrosis*. London: Chapman and Hall, 1994; 99–129.
2. Crystal RG. Oxidants and respiratory tract epithelial injury: pathogenesis and strategies for therapeutic intervention. *Am J Med* 1991; **91 (suppl3c)**: 39S–44S.
3. Goldstein W, Döring G. Lysosomal enzymes and proteinase inhibitors in the sputum of patients with cystic fibrosis. *Am Rev Respir Dis* 1986; **134**: 49–56.
4. Meyer KC, Zimmerman J. Neutrophil mediators. Pseudomonas and pulmonary dysfunction in cystic fibrosis. *J Lab Clin Med* 1993; **121**: 654–61.
5. Regelmann WE, Sieferman CM, Herron JM, Elliott GR, Glawson CC, Gray BH. Sputum peroxidase activity corrrelates with the severity of lung disease in cystic fibrosis. *Pediatr Pulmonol* 1995; **19**: 1–9.
6. Koller DY, Götz M, Eichler I, Urbanek R. Eosinophilic activation in cystic fibrosis. *Thorax* 1994; **49**: 496–499.
7. Roum JH, Buhl R, McElvany NG, Borok Z, Crystal RG. Systemic deficiency of glutathione in cystic fibrosis. *J Appl Physiol* 1993; **75**: 2419–2424.
8. Portal BC, Richard MJ, Faure HS, Hadjian AJ, Favier AE. Altered antioxidant status and increased lipid peroxidation in children with cystic fibrosis. *Am J Clin Nutr* 1995; **61**: 843–847.
9. Yoo JH, Erzurum SC, Hay JG, Lemarchand P, Crystal RG. Vulnerability of the human airway epithelium to hypoxia. Constitutive expression of the catalase gene in human bronchial epithelial cells despite oxidant stress. *J Clin Invest* 1994; **93**: 297–302.
10. Matheson NR, Wong PS, Travis J. Enzymatic inactivation of human alpha 1-proteinase inhibitor by neutrophil myeloperoxidase. *Biochem Biophys Res Commun* 1979; **88**: 402–409.
11. Koyama S, Kobayashi T, Kubo K, Sekiguchu M, Ueda G. Recombinant-human superoxide dismutase attenuates endotoxin-induced lung injury in awake sheep. *Am Rev Respir Dis* 1992; **145**: 1404–1409.
12. Sies H. Antioxidant activity in cells and organs. *Am Rev Respir Dis* 1987; **136**: 478–80.
13. Leff JA, Wilke CP, Hybertson BM, Shanley PF, Beehler CJ, Repine JE. Postinsult treatment with N-Acetyl-L-cysteine decreases IL-1 induced neutrophil influx and lung leaks in rats. *Am J Physiol* 1993; **265**: L501–L506.
14. Thibeault DW, Rezaiekhaligh M, Mabry S. Prevention of chronic pulmonary oxygen toxicity in young rats with liposome-encapsulated catalase administrated intratracheally. *Pediatr Pulmonol* 1991; **11**: 318–327.

### Cytokines proinflammatoires et leurs inhibiteurs

*Jean-Michel Dayer*
Division of Immunology and Allergy, University Hospital, Geneva, Switzerland

Concomittant à la réaction inflammatoire médiée par des substances pro-inflammatoires (cytokines telles que l'IL-1 et le TNFα), l'organisme utilise des mécanismes naturels pour limiter la propagation de l'inflammation. Parmi eux sont: (1) les antagonistes spécifiques au niveau des récepteurs des cytokines (IL-1Ra); (2) les cytokines ayant une activité biologique s'opposant aux cytokines pro-inflammatoires (ex: IL-4, Il-13, IL-10, TGFβ); (3) les inhibiteurs agissant en tant que protéine liant le ligand (ex.: récepteurs solubles des cytokines) ou des protéines plasmatiques agissant en tant que protéines porteuses; (4) les inhibiteurs d'enzymes induisant la conversion de procytokines inactives en cytokines actives; (5) les enzymes scindant les récepteurs membranaires des cytokines; (6) la diminution de l'expression des récepteurs cytokinaires; (7) les enzymes dégradant les cytokines pro-inflammatoires; (8) les auto-anticorps naturels anti-cytokines. L'inhibition peut aussi survenir à d'autres niveaux tels que: (1) l'inhibition du stimulus induisant la production des molécules pro-inflammatoires (ex.: anticorps anti-CD14) en relation avec la stimulation par les lipoplysaccharides (endotoxines), anticorps anti-CD 11 et anti-CD 19 en relation avec les interactions lymphocytes-monocytes pour la production d'IL-1, de TNFα, de PGE2 et de métalloprotéases; (2) l'inhibition au niveau de la transduction, de la transcription et de la traduction; (3) inhibition au niveaude la maturation et de la sécrétion des cytokines; (4) inhibition par la modification de la structure fonctionnelle des cytokines (mutéines).

Les facteurs devant être considérés dans le cadre du concept de l'équilibre entre cytokines et inhibiteurs de cytokines sont (1) la séquence d'apparition de ces molécules, (2) le fait que leur quantité relative est plus importante que leur quantité absolue; (3) l'effet bénéfique potentiel pouvant exister, pendant une courte période, pour une cytokine pro-inflammatoire dans le rôle de la défense de l'hôte (ex.: TNFα); (4) la complexité de certains systèmes cytokinaires tels que le système IL-1 qui possède au moins deux types d'antagonistes naturels (IL-1Ra, Il-1SR) menant au paradoxe de la liaison de deux inhibiteurs entre eux, qui permet à la molécule agoniste (IL-1) d'exercer toute son activité; (5) la formation de complexes instables de cytokines/inhibiteurs de cytokines. Ainsi, le concept d'inhibiteurs de cytokines doit être considéré plus dans le cadre d'un système 'tampon' que d'une réaction 'on/off'.

Enfin, un autre aspect important, lors de l'analyse de l'impact d'inhibiteurs de cytokines, est celui de la dissociation entre leurs effets immunosuppresseurs et anti-inflammatoires. Par exemple, la protéine de fusion TNF-R: Fc est 100 fois plus puissante pour inhiber les métalloprotéases et les PGE2 produites par les fibroblastes que pour inhiber la prolifération des lymphocytes T et la production de leurs cytokines. Par conséquent, les futures approches thérapeutiques doivent bien distinguer lesquels parmi les inhibiteurs des cytokines ont un effet à prédominance anti-inflammatoire, anti-activation des systèmes protéolytiques et immunosuppresseurs.

### Références

1. Arend WP, Dayer JM. Inhibition of the production and effects of IL-1 and TNFα in rheumatoid arthritis. *Arthritis Rheum* 1995; **38**: 151–160.
2. Burger D, Dayer JM. Inhibitory cytokines and cytokine inhibitors. *Neurology* 1995; **45 (suppl.6)**: S39–S43.
3. Nicod LP, Galve de Rochemonteix B, Dayer JM. Modulation of Il-1 receptor antagonist and TNF-soluble receptors produced by alveolar macrophages and blood monocytes. In: *Annals of the New York Aademy of Sciences*, Vol. **725**, 1994, pp. 323–330.
4. Dayer JM, Arend WP. Cytokines and growth factors. In: Kelley WN, Harris ED, Ruddy S, Sledge CB, eds. *Textbook of Rheumatology*, 5th edn. Philadelphia: Saunders (in press).
5. Isler P, Vey E, Zhang JH, Dayer JM. Cell surface glycoproteins expressed on actived human T cells induce production of interleukin-1 beta by monocytic cells: a possible role of CD69. *Eur Cytokine Netw* 1993; **4**: 15–23.
6. Lacraz S, Isler P, Vey E, Welgus HG, Dayer JM. Direct contact between T lymphocytes and monocytes is a major pathway for induction of metalloproteinase expression. *J Biol Chem* 1994; **269**: 22027–22033.
7. Burger D, Chicheportiche R, Giri J, Dayer JM. The inhibitory activity of human interleukin-1 receptor antagonist is enhanced by type II interleukin-1 soluble receptor and hindered by type I interleukin-1 soluble receptor. *J Clin Invest* 1995; **96**: 38–41.
8. Nicod LP, El Habre F, Dayer JM, Boerhinger N. Il-10 decreases tumor necrosis factor a and ß in alloreactions induced by human lung dendritic cells and macrophages. *Am J Respir Cell Mol Biol* 1995; **13**: 83–90.
9. Lacraz S, Nicod LP, Chicheportiche R, Welgus HG, Dayer JM. IL-10 Inhibits metalloproteinase and stimulates TIMP-1 production in human mononuclear phagocytes. *J Clin Invest* 1995; **96**: 2304–2310.

### Les cytokines de la manifestation pulmonaire de la mucoviscidose

*Melvin Berger, Tracy Bonfield, James Panuska et Michael Konstan*
Departments of Pediatrics and Medicine, Case Western Reserve University School of Medicine, Cleveland, Ohio, USA

Plusieurs caractéristiques de la mucoviscidose telles que l'afflux excessif de neutrophiles vers les poumons, la cachexie et l'hyperglobulinémie, suggèrent une implication de cytokines pro-inflammatoires, par exemple l'interleukine (IL)-1, le facteur nécrosant des tumeurs (TNF), l'IL-8, et l'IL-6. Les concentrations et l'action de ces cytokines étant plus importante au site de leur production qu'au niveau systémique, nous avons recherché leur présence au niveau même du poumon, dans des échantillons de fluide de lavage bronchoalvéolaire (LBA) et de prélèvements obtenus par brossage des voies respiratoires.[1]

Les concentrations des trois cytokines pro-inflammatoires IL-1, IL-6 et TNF étaient considérablement élevées dans les fluides BAL provenant de patients atteints de mucoviscidose par rapport à ceux provenant de témoins sains. Alors que l'IL-8 n'est pas détectable dans le fluide de LBA des témoins sains, la concentration de ce puissant facteur chimiotactique pour les neutrophiles atteint 30 ng/ml, et ce même au cours d'une période stable et éloignée d'une exacerbation de la maladie pulmonaire. Des concentrations modérément élevées de l'antagoniste du récepteur de l'IL-1 et des formes solubles des récepteurs du TNF ont été également retrouvées dans le fluide BAL de patients CF, mais le rapport inhibiteur de cytokine/cytokine était très faible. L'IL-10, une cytokine régulatrice exerçant un effet suppresseur sur la production des cytokines pro-inflammatoires, n'était pas retrouvée dans les échantillons CF ou à des taux fortement réduits par rapport à celui de 3,5 ng/ml observé dans les LBA des sujets sains. Ces différences étaient plus marquées chez des patients atteints de mucoviscidose et infectés par *Pseudomonas* par rapport à ceux infectés par *Haemophilus influenzae* ou *Staphyloccocus aureus*. Chez un nourrisson CF infecté, un traitement antibiotique intensif a entrainé une diminution des concentrations en IL-6 et IL-8, sans toutefois observer leur retour à la normale. En revanche l'IL-10 est restée indétectable avant et après le traitement aux antibiotiques.

Afin de comprendre les mécanismes de cette modulation des cytokines pro-inflammatoires, nous avons exploré les diverses sources cellulaires possibles de ces cytokines au sein du poumon CF. Les macrophages présents dans les échantillons de LBA des patients CF présentent une immunofluorescence positive pour l'IL-1, l'IL-6, l'IL-8 et le TNF. Ce même profil est obtenu dans des macrophages isolés des LBA de sujets témoins, mais incubés *in vitro* avec du lipopolysaccharide (LPS) avant la coloration. En revanche, les macrophages provenant des témoins non traités par le LPS sont majoritairement négatifs en immunofluorescence. Fait inattendu, les macrophages isolés des patients CF présentent une immunofluorescence positive pour l'IL-10 et il en est de même pour les macrophages isolés de témoins sains traités par le LPS.

D'autres études visant à déterminer la source de l'IL-10 retrouvée dans le fluide de lavage alvéolaire des témoins sains ont montré que des cellules épithéliales bronchiques obtenues par brossage étaient positives pour l'IL-10 en immunofluorescence, possédaient des ARNm IL-10, et sécrétaient de l'IL-10 lorsqu'elles étaient placées en culture primaire *in vitro*. Les cellules broncho-épithéliales des patients CF renfermaient nettement moins d'IL-10 en immunofluorescence.[2] Ces données suggèrent que dans le poumon normal les cellules broncho-épithéliales produisent la cytokine anti-inflammatoire IL-10 afin de réguler de façon négative les réponses locales inflammatoires et de prévenir les lésions de l'épithélium. Dans la mucoviscidose ce mécanisme suppresseur est défectueux dans le poumon infecté. En revanche, dans le cas de la mucoviscidose, les cellules broncho-épithéliales produisent activement de l'IL-6 et de l'IL-8, qui exacerbent à leur tour le processus inflammatoire.

A la lumière de ces observations, les cellules broncho-épithéliales pourraient jouer dans le poumon un rôle déterminant dans la régulation locale des processus immunologiques et inflammatoires. Cette hypothèse soulève à son tour les questions suivantes: une fonction CFTR défective pourrait-elle altérer la production de cytokines des cellules épithéliales, même en l'absence d'infection? Les réponses des cellules épithéliales CF aux stimuli pouvant réguler de façon positive la production de l'IL-8 et/ou réguler de façon négative la production de l'IL-10 seraient-elles différentes des réponses des cellules normales?

### Références

1. Bonfield TL, Panuska JR, Konstan MW, *et al.* Inflammatory cytokines in cystic fibrosis lungs. *Am J Respir Crit Care Med* 1995; **152**: 2111–2118.
2. Bonfield TL, Konstan MW, Burfiend P, Panuska JR, Hilliard JB, Berger M. Normal bronchial epithelial cells constitutively produce the anti-inflammatory cytokine interleukin-10, which is downregulated in CF. *Am J Respir Cell Mol Biol* 1995; **13**: 257–261.

### Régulation du gène CFTR par les agents anti-inflammatoires

*Aleksander Edelman, Françoise Besançon* et Marie-Yvonne Baudouin-Legros*

INSERM U.323, CHU Necker and *INSERM U417, CHU St Antoine, Paris, France

La mucoviscidose résulte de mutations du gène de 250 kb qui code pour la protéine CFTR, un canal $Cl^-$ régulé par l'AMP cyclique. La protéine CFTR se situe à la membrane apicale des cellules épithéliales sécrètoires.[1,2] Les mutations du gène CFTR provoquent des anomalies de synthèse, du routage, ou de la fonction de la protéine CFTR.[3] La maladie a de multiples manifestations dominées par les anomalies de l'épithélium de surface des voies aériennes qui sécrète en grandes quantités un mucus épais favorisant l'inflammation et les infections chroniques.

L'inflammation provoque la formation d'un grand nombre de médiateurs intracellulaires et tissulaires capables de modifier les fonctions cellulaires. Ainsi les interférons (IFN), une famille de protéines aux multiples fonctions produites en réponse aux infections virales et bactériennes, sont des médiateurs majeurs de la défense de l'hôte contre les agents infectieux.[4] IFNα et IFNβ sont synthétisés principalement par les lymphocytes et les fibroblastes (respectivement). IFNγ, une cytokine clé du réseau immunitaire, est synthétisée par les lymphocytes T et les cellules 'natural killer' après stimulation antigénique (2) et son rôle crucial dans la défense contre les infections parasitaires et virales a récemment été mis en lumière.[5] Un autre médiateur de l'inflammation, le *Tumor Necrosis Factor* α (TNFα), est synthétisé par les monocytes/macrophages aussi bien que par les cellules B et T, les cellules de Kupffer, les cellules gliales et les adipocytes,[6] et entraine de multiples effets intracellulaires. IFNγ et TNFα modifient également le transport ionique transépithélial, mais les mécanismes responsables de cette action ne sont pas bien définis.

La colonisation bactérienne et les médiateurs tissulaires de l'inflammation pourraient tous les deux altérer les fonctions cellulaires. Cependant leur influence directe ou indirecte sur la fonction de CFTR n'a pas été clairement définie. Ceci nous a conduit à explorer les effets des IFN et du TNFα sur l'expression du gène CFTR.

Dans la mesure où l'inflammation et les infections pulmonaires constituent le problème majeur de la mucoviscidose, les antibiotiques et les médicaments anti-inflammatoires sont souvent administrés de façon systématique chez ces patients. L'étude de la littérature montre que ces molécules pourraient modifier les fonctions des cellules épithéliales. C'est la raison pour laquelle nous avons étudié les actions exercées par deux médicaments anti-inflammatoires différents (aspirine et dexamethasone) sur l'expression du gène CFTR.

Deux lignées cellulaires adénocarcinomateuses (HT29 et T84) connues pour exprimer de grandes quantités de protéine CFTR nous ont servi de modèles expérimentaux. Nous avons observé que l'IFNγ à faibles concentrations (de 0.1 à 100 UI/ml), diminue l'expression du gène CFTR (diminution des ARN messagers et de la protéine CFTR) par un mécanisme dose-dépendant et post-transcriptionnel. Cet effet était dépendant du temps: la diminution des ARN messagers induite par l'IFNγ était significative après 3 heures d'incubation et maximale à 12 heures. IFNγ et β étaient inactifs sur l'expression du gène CFTR dans les cellules T84, suggérant une certaine spécificité de l'IFNγ. L'inhibition de l'expression du gène CFTR induite par l'IFNγ entrainait aussi une diminution de fonction: le traitement par IFNγ réduisait les flux $Cl^-$ dépendants de l'AMPc. Le TNFα, qui est produit de façon concomittante avec l'IFNγ durant les réactions inflammatoires et immunitaires diminuait aussi l'expression du gène CFTR, et de façon synergique avec l'IFNγ[9]

Ces résultats suggèrent que les processus inflammatoires pourraient augmenter la symptomatologie de la mucoviscidose et diminuer l'efficacité d'une thérapie génique. C'est pourquoi nous étudions, pour prévenir l'action négative de ces deux cytokines, les effets sur l'expression du gène CFTR de deux molécules appartenant à des familles différentes d'anti-inflammatoires: la dexamethasone comme molécule stéroidienne et l'aspirine comme molécule non stéroidienne. Dans nos conditions expérimentales, la dexamethasone (1μ mol/l) ne provoque aucun effet, ni au niveau des ARN messagers ni au niveau fonctionnel. Au contraire, le traitement pendant 48 heures des cellules T84 par l'aspirine ou par l'acide salicylique (0.5 à 1 mM) diminue les quantités de transcript CFTR et les flux $Cl^-$ dépendants de l'AMPc. L'effet de l'aspirine ne nécessite pas la synthèse *de novo* de la protéine, puisqu'il persiste après le traitement des cellules par cycloheximide. Cet effet n'est pas dû à l'activation de l'adényl cyclase puisque la production d'AMPc induite par la forskoline n'est pas modifiée par le traitement des cellules par l'aspirine (ou l'acide salicylique).

Ces résultats suggèrent que la production d'IFNγ et de TNFα en réponse aux infections pourrait aggraver les dysfonctions pulmonaires chez les sujets atteints de mucoviscidose, et créer un phénotype localisé de mucoviscidose chez les patients non-mucoviscidosiques mais atteints d'une maladie pulmonaire chronique bactérienne ou inflammatoire. De plus, nos résultats mettent en évidence une nouvelle action de l'aspirine, utilisée communément comme anti-inflammatoire dans la mucoviscidose. De fortes doses d'aspirine prises de façon chronique pourraient également diminuer la sécrétion de chlore liée à CFTR et aggraver ainsi l'état clinique des patients.

### Références

1. Quinton, P. Cystic fibrosis: a disease in electrolyte transport. *FASEB J* 1990; **4**: 2709.

2. Fuller C, Benos DJ. CFTR! *Am J Physiol* 1992; **263**: C267.

3. Welsh JM, Smith AE. Molecular mechanisms of CFTR chloride channel dysfunction in cystic fibrosis. *Cell* 1993; **73**: 1251.

4. De Maeyer E, De Maeyer-Guignard J. *Interferons and Other Regulatory Cytokines.* New York: John Wiley, 1988.
5. Dalton DK, *et al.* Multiple defects of immune cell function in mice with disrupted interferon-γ genes. *Science* 1993; **259**: 1739.
6. Lewis SA, *et al.* Modulation of epithelial permeability by extracellular macromolecules. *Physiol Rev* 1995; **75**: 561.
7. Besançon F, *et al.* Interferon-γ downregulates CFTR gene expression in epithelial cells. *Gastroenterology* 1995; **109**: 639.

### Traitement anti-inflammatoire dans la mucoviscidose: bases théoriques et prospectives

*Michael W. Konstan et Melvin Berger*
Department of Pediatrics, Case Western Reserve University School of Medicine, Cleveland, Ohio, USA

L'accumulation des neutrophiles dans l'arbre respiratoire est à l'évidence excessive et joue un rôle important dans la destruction des poumons qui reste la première cause de morbidité et de mortalité dans la mucoviscidose.[1] Les mécanismes selon lesquels le neutrophile peut altérer la fonction pulmonaire ou ses mécanismes de défense sont multiples et exposés en détail par d'autres orateurs dans ce symposium. Plusieurs études récentes[2,3] dont la nôtre qui concerne des patients avec une atteinte modérée[4] permettent d'affirmer clairement que l'emballement du processus inflammatoire commence très tôt dans l'évolution de la mucoviscidose et persiste inéluctablement avec des accès d'intensification brutale.[5]

L'infection pulmonaire chronique est sans nul doute un stimulus majeur de cette réponse inflammatoire et les exacerbations infectieuses augmentent le nombre de polynucléaires neutrophiles et leurs effets délétères. Certaines observations récentes semblent montrer que l'inflammation précède l'infection[2,3] – et/ou que la régulation des réactions inflammatoires dans le poumon pourrait être génétiquement anormale dans la mucoviscidose, même si le rôle des exacerbations infectieuses dans l'intensification des lésions dues à l'inflammation ne peut être nié.

D'une claire évidence, le traitement visant à diminuer les lésions inflammatoires doit également inclure la prévention ou la diminution du degré de l'infection. Cependant, une fois que *Pseudomonas* commence à être implanté, il est rare de pouvoir l'éradiquer et ce malgré l'apparition incessante d'antibiotiques plus actifs. L'usage de bronchodilatateurs, l'administration intrabronchique de la rh-DNase et la pratique régulière des techniques de désencombrement des voies aériennes contribuent à atteindre l'objectif principal de la préservation du capital respiratoire; malgré ces efforts, le processus inflammatoire se poursuit et amène inexorablement la dégradation de l'état des patients. Pour ces raisons, de nouvelles approches thérapeutiques sont proposées ayant pour but soit une action anti-inflammatoire, soit un rôle plus direct s'opposant au défaut primaire telles que l'Amiloride et/ou l'UTP voire même la thérapie génique.

Dans le champ d'action des thérapeutiques anti-inflammatoires, il est clair que la cible demeure le polynucléaire. La première étape de la migration du leucocyte dans le poumon implique une activation et une attraction de ces cellules par des médiateurs solubles. De nombreuses études ont permis d'établir le contenu des sécrétions pulmonaires dans la mucoviscidose – dont la forte teneur de tous les principaux groupes de facteurs chimiotactiques du polynucléaire et notamment LTB4, C5a et IL8 (revus dans 1). Il est de ce fait improbable que les molécules antagonistes de ces facteurs, capables de s'opposer à leur production ou de bloquer leur récepteur, aient une activité notable. D'autre part, on ne dispose pas, même à la phase des essais, d'agent pharmacologique qui pourrait interférer avec des molécules d'adhésion qu'utilise le neutrophile lors de son roulement sur les capillaires sanguins et de sa diapédèse pour migrer dans l'épithélium pulmonaire.

En raison de leurs effets secondaires prévisibles qui se sont révélés d'ailleurs problématiques lors d'un essai à grande échelle aux USA,[6] les glucocorticodes nous ont paru devoir céder la place aux anti-inflammatoires non stéroidiens (AINS), en particulier l'Ibuprofen. Ce médicament agit directement sur le neutrophile dont il diminue à la fois les capacités de migration et de dégranulation, ainsi qu'à fortes doses nécessaires mais atteignables en clinique, il bloque la libération de LTB4 ainsi que l'activité de la cyclooxygenase (revu dans 7). A l'aide d'un modèle simulant l'infection intra-bronchique des poumons – à savoir la distribution du polynucléaire dans les cryptes gingivales au niveau buccal – nous avons pu montrer que l'Ibuprofen interfère avec la mobilisation du polynucléaire chez le volontaire sain tout autant que chez les patients atteints de mucoviscidose.[8]

Dans un modèle expérimental de l'infection chronique à *Pseudomonas* nous avons pu montrer que l'Ibuprofen réduit la surface d'extension de l'inflammation sans toutefois augmenter le rôle nocif des microorganiques infectieux.[7] Après les premières études qui ont permis de décrire la pharmacocinétique de l'Ibuprofen chez les patients atteints de mucoviscidose, nous avons mis en place un essai sur 4 ans avec ce médicament chez les patients présentant une atteinte pulmonaire modérée. Comparé à un groupe placebo, les patients traités par l'Ibuprofen présentent de manière significative un ralentissement de la dégradation de leur fonction pulmonaire et du score radiologique de Brasfield, conservent leur niveau de poids par rapport à leur poids idéal et ont une tendance a être moins souvent hospitalisés.[9] Cette action est plus prononcée dans le groupe des plus jeunes patients (5–13 ans): leur taux annuel de déclin du VEMS a été plus lent (88% de réduction de la vitesse dans le groupe traité). Il n'y a pas eu d'effets secondaires observés.

L'usage chez l'homme de l'Ibuprofen est maintenant répandu partout aux USA et dans les autres parties du monde. Il est nécessaire de fixer la posologie en fonction des caractéristiques cinétiques propres des patients. D'autres AINS sont à l'essai avec des résultats tout aussi prometteurs (revus dans 10). Les rapports montrant que les stigmates de l'inflammation sont retrouvés précocement même chez le nourrisson dans le cadre de la mucoviscidose, joints à nos observations d'une meilleure réponse à l'Ibuprofen des sujets les plus jeunes, incitent à tester l'Ibuprofen chez le tout jeune enfant. D'ici à ce que la thérapie génique soit mise en place, les médicaments anti-inflammatoires verront certainement leur part augmenter pour atteindre une place importante dans l'arsenal thérapeutique de la mucoviscidose.

### Références

1. Konstan MW, Berger M. Infection and inflammation in the lung in cystic fibrosis. In: PB Davis, ed, *Cystic Fibrosis*. New York: Marcel Dekker, 1993.
2. Kahn TZ, Wagener JS, Bost T, Martinez J, Accurso FJ, Riches DW. Early pulmonary inflammation in infants with cystic fibrosis. *Am J Respir Crit Care Med* 1995; **151**: 1075–82.
3. Armstrong DS, Grimwood K, Carzino R, Carlin JB, Olinsky A, Phelan PD. Lower respiratory infection and inflammation in infants with newly diagnosed cystic fibrosis. *BMJ* 1995; **310**: 1571–1572.
4. Konstan MW, Hilliard KA, Norvell TM, Bergen M. Bronchoalveolar lavage findings in cystic fibrosis patients with stable, clinically mild lung disease suggest ongoing infection and inflammation. *Am J Respir Crit Care Med* 1994; **150**: 448–454.
5. Cantin A. Cystic fibrosis lung inflammation: early, sustained and severe. *Am J Respir Crit Care Med* 1994; **150**: 448–454.
6. Eigen H, Rosenstein BJ, FitzSimmons S, Schidlow DV. A multicenter study of alternate-day prednisone therapy in patients with cystic fibrosis; Cystic Fibrosis Foundation Prednisone Trial Group. *J Pediatr* 1994; **126**: 515–523.
7. Konstan MW, Vargo KM, Davis PB. Ibuprofen attenuates the inflammatory response to *Pseudomonas aeruginosa* in a rat model of chronic pulmonary infection: Implications for anti-inflammatory therapy in cystic fibrosis. *Am Rev Respir Dis* 1990; **141**: 186–192.
8. Konstan MW, Hilliard KA, Davis PB. Effect of ibuprofen on neutrophil delivery to mucosal surfaces. *Pediatr Pulmonol Suppl* 1989; **4**: 152–153.
9. Konstan MW, Byard PJ, Hoppel CL, Davis PB. Effect of high-dose ibuprofen in patients with cystic fibrosis. *N Engl J Med* 1995; **332**: 848–854.
10. Konstan MW. Anti-inflammatory therapy in cystic fibrosis. In: *New Insights into Cystic Fibrosis*. New Jersey: Gardiner Caldwell SynerMer, Vol 3 (2), 1995.

### Les différentes approches pour la thérapie génique de la mucoviscidose

*Patricia Lemarchand*
INSERM U25, Faculté de Médecine Necker-Enfants Malades, Paris, France

L'identification du gène responsable de la mucoviscidose (ou gène CFTR) a ouvert la porte à la thérapie génique pour traiter les manifestations respiratoires de la mucoviscidose. La faisabilité du transfert de gène dans les cellules épithéliales a d'abord été démontrée dans des cultures d'épithélium bronchique de sujets atteints de mucoviscidose. Quand le gène normal CFTR est transféré *in vitro* dans les cellules épithéliales provenant d'un individu atteint, les anomalies biologiques cellulaires associées à la mucoviscidose sont corrigées. Il y a donc une 'complémentation' du gène endogène anormal par le gène transféré. Ces premières expériences de complémentation *in vitro* ont conduit à la recherche de vecteurs pour délivrer le gène CFTR dans les voies aériennes. Le vecteur idéal de transfert du gène CFTR *in vivo* devrait délivrer le gène dans les cibles cellulaires appropriées sans provoquer de toxicité ni d'inflammation.

Bien qu'aucun des vecteurs disponibles à l'heure actuelle ne possède ces critères, des études ont démontré dans de nombreux modèles animaux la faisabilité du transfert du gène CFTR dans le poumon, et des essais cliniques chez l'homme ont débuté, utilisant les adénovirus recombinants et les liposomes comme vecteurs de transfert du gène CFTR dans l'épithélium des voies aériennes. Cependant la physiopathologie de la mucoviscidose est peu claire et de nombreuses questions restent à résoudre pour une thérapie génique efficace de la mucoviscidose. Tout d'abord au niveau cellulaire, CFTR joue un rôle important dans plusieurs fonctions cellulaires. Bien que les manifestations cliniques respiratoires de la mucoviscidose se situent dans les voies aériennes, il n'est pas possible d'évaluer les fonctions de CFTR de façon reproductible dans l'épithélium bronchique *in vivo*. Il est donc difficile de démontrer au site majeur des manifestations cliniques de la maladie qu'une stratégie de thérapie génique corrige réellement les déficits fonctionnels associés aux mutations du gène CFTR, y compris les troubles de la conductance $Cl^-$ et l'hyperabsorption de $Na^+$. Deuxièmement, CFTR a été localisée à la fois dans l'épithélium de surface et dans les glandes sous-muqueuses des voies aériennes. Aucun des vecteurs utilisés actuellement dans les essais cliniques ne permet un transfert de gènes efficace dans les glandes sous-muqueuses. De même, si la fonction CFTR

dans les glandes sous-muqueuses est nécessaire pour rétablir une clairance normale du mucus dans les voies aériennes, les approches actuelles ne pemettront probablement pas d'avoir un impact sur la maladie dans les bronches cartilagineuses. Enfin, le caractère relativement bénin de la maladie pulmonaire chez les sujets portant un allèle codant pour une molécule CFTR partiellement défective, suggère qu'une reconsitution génétique complète ne sera pas nécessaire. Cependant, le transfert de gènes dans l'épithélium des voies aériennes chez le primate tend à être d'efficacité faible et hétérogène, même avec les vecteurs adénoviraux recombinants.

En résumé, plusieurs questions clés doivent être résolues pour que la thérapie génique de la mucoviscidose soit efficace. A côté du transfert du gène CFTR, des stratégies de thérapie génique 'non-spécifique' qui pourraient être appliquées aux sujets atteints de mucoviscidose ont été développées, notamment la protection contre le stress oxydant et l'augmentation de la durée de survie du greffon pulmonaire.[1–3]

*Thérapie génique anti-oxidative pour la mucoviscidose*

Une partie importante des lésions dans la mucoviscidose est due aux oxydants relargués par les cellules inflammatoires à la surface de l'épithélium des voies aériennes, en particulier les neutrophiles. Le nombre de neutrophiles dans le lavage alvéolaire de sujets atteints de mucoviscidose étant 100 à 1000 fois supérieur à celui des gens normaux, la quantité potentielle des oxydants dans l'épithélium des voies aériennes est énorme. La balance oxydants-antioxydants est encore plus en faveur des oxydants en raison des niveaux de glutathion très diminués chez les sujets atteints de mucoviscidose. Une stratégie pour protéger l'épithélium est d'augmenter la couverture anti-oxydante de la surface épithéliale, permettant ainsi aux cellules inflammatoires sur la surface épithéliale de phagocyter et de tuer les bactéries, mais protégeant l'épithélium des lésions induites par les oxydants relargués par les phagocytes durant la phagocytose. Des études préliminaires *in vitro* ont montré que le transfert de gène de la catalase humaine pouvait protéger les cellules épithéliales bronchiques contre le stress oxydant.

*Amélioration de la survie du greffon pulmonaire par thérapie génique*

La transplantation pulmonaire et cardio-pulmonaire représente un espoir thérapeutique nouveau pour les patients atteints de mucoviscidose. Malgré les moyens thérapeutiques conventionnels, de nombreuses complications fréquentes et graves menacent la fonction du greffon, notamment l'oedème de réimplantation responsable d'une défaillance respiratoire aiguë chez 20% des patients avec une mortalité immédiate importante. Cet oedème est la conséquence du syndrome d'ischémie-reperfusion pulmonaire. L'augmentation de la durée de survie du greffon par l'utilisation de la thérapie génique constitue une nouvelle approche thérapeutique prometteuse. Le transfert du gène codant pour la superoxyde dismutase et de celui codant pour la catalase humaine pourrait protéger l'endothélium vasculaire pulmonaire contre l'ensemble des oxydants produits lors de la reperfusion du greffon. Les vecteurs adénoviraux étant efficaces pour transférer des gènes *in vivo* dans la vascularisation pulmonaire, nous avons évalué la faisabilité du transfert de gène dans le greffon pulmonaire dans un modèle d'allotransplantation pulmonaire chez le porc. Les vecteurs adénoviraux peuvent être utilisés pour transférer des gènes *in vivo* dans le greffon pulmonaire, mais de nombreux paramètres limitent l'eficacité du transfert de gène et nécessiteront d'être améliorés avant qu'une application clinique puisse être envisagée.

### Références

1. Alton EWFW, Geddes DM. Gene therapy for cystic fibrosis: a clinical perspective. *Gene Therapy* 1995; **2**: 88–95.
2. Wilson JM. Gene therapy for cystic fibrosis: challenges and futures directions. *J Clin Invest* 1995; **96**: 2547–2554.
3. Rosenfeld MA, Collins FS. Gene therapy for cystic fibrosis. *Chest* 1996; **109**: 241–252.

### Thérapie génique dans la mucoviscidose

*Gabriel Bellon et Andrea Pavirani**

Hospices Civils de Lyon, Unité 'Pneumologie Pédiatrique-Mucoviscidose' Centre Hospitalier Lyon Sud, 69310 Pierre Bénite et *Transgène SA, 11 rue de Molsheim, 67082 Strasbourg, France

L'état actuel des connaissances laisse penser que la thérapie génique sera un moyen de traiter, ou au moins de prévenir les manifestations respiratoires de la mucoviscidose (CF pour *Cystic Fibrosis*). Les études précliniques démontrent que l'Ad-CFTR, adénovirus déficient pour la réplication exprimant le gène CFTR humain normal (*cystic fibrosis transmembrane conductance regulator*), est capable de transférer de façon efficace le gène CFTR dans des cellules épithéliales respiratoires de patients atteints de CF en culture, ou dans les voies respiratoires des animaux de laboratoire.

Le but de cet essai clinique a été de tester chez 6 patients atteints de CF la tolérance et l'efficacité biologique d'une dose unique d'un virus déficient pour la réplication exprimant le gène CFTR humain normal administré par instillation directe au niveau de la muqueuse nasale le premier jour, et par voie d'aérosol au niveau du tractus respiratoire inférieur le lendemain. Les doses administrées ont été par groupes de 2 patients, $10^5$ *pfu* (pour *plaque forming units*), $10^7$ *pfu* et $4.10^8$ au niveau du nez, et $10^7$, $10^8$ et $5{,}4.10^8$ au niveau bronchique.

Il n'a pas été observé d'effet toxique aigu, d'augmentation du titre des Ac anti-adénovirus, de diffusion ou d'excrétion de particules de vecteur infectieux. Chez un patient, du DNA de l'Ad-CFTR a été dépisté dans le sang et les urines 2 jours après l'aérosol.Le DNA de l'Ad-CFTR a été mis en évidence jusqu'à J 21 au niveau du brossage nasal, J 15 au niveau du brossage bronchique et de la salive, J 8 dans le lavage broncho-alvéolaire (LBA) et J 4 au niveau du brossage amygdalien. Du RNA messager (mRNA) CFTR (détecté par RT-PCR) et la protéine CFTR (détectée par immunohistochimie) ont été mis en évidence sur brossage nasal et bronchique jusqu'au quinzième jour après l'administration.

Ces résultats montrent que la nébulisation d'un vecteur adénoviral est une approche possible pour le traitement préventif des manifestations respiratoires de la mucoviscidose.

REMERCIEMENTS. Ce travail a été financé par les Hospices Civils de Lyon, le Ministère de la Santé, l'AFLM, le Ministère de la Recherche, la DRASS Rhône-Alpes et l'Association Expector.

### Inflammation pulmonaire associée aux transferts de gènes

*Claire Danel*

Service d'Anatomie Pathologique, Hopital Laënnec, Paris, France

Pour qu'une thérapie génique soit efficace, l'information génétique doit être transmise et exprimée dans les cellules cibles. De nombreux vecteurs tels que les rétrovirus, les adénovirus, les virus associés aux adénovirus ou bien encore les liposomes ont été utilisés pour optimiser ce processus. Les vecteurs adénoviraux présentent un certain nombre d'avantages.[7] Leur faculté à transfecter du matériel génétique efficacement dans des cellules épithéliales respiratoires les a fait choisir pour les premiers essais de thérapie génique sur la mucoviscidose (CF).[2,3,5,7–12] Le succès de cette démarche dépend en grande partie non seulement de l'intensité et de la durée de l'expression transgénique, mais également de son innocuité. De nombreuses questions demeurent concernant la toxicité de l'administration de vecteurs adénoviraux dans les poumons de patients atteints de CF. En théorie, plusieurs problèmes peuvent survenir lors de l'utilisation d'adénovirus recombinants, tels l'induction d'une immunité cellulaire, une éventuelle recombinaison avec un virus sauvage, la production d'anticorps neutralisants provoquant une réduction de l'efficacité du transfert lors de réintroductions ultérieures, et une inflammation dans le territoire d'administration du virus avec d'éventuelles répercussions systémiques. Pour tenter de répondre à un certain nombre de ces questions, des études animales ont été effectuées, en particulier sur des primates et des 'cotton rats'. Ces études sont importantes pour évaluer la toxicité potentielle de ces virus recombinants sur l'épithélium respiratoire et le parenchyme pulmonaire, en particulier l'adénovirus contenant l'ADN recombinant du CFTR humain (AdCFTR).

### *Les inflammations pulmonaires chez l'animal*

Le 'cotton rat' semble être un bon modèle pour l'évaluation des répercussions pulmonaires de l'administration des adénovirus recombinants.[6] Des études ont montré que chez 'le cotton rat' on observe une bonne transfection de ces virus dans les cellules épithéliales de l'arbre bronchique jusqu'aux bronchioles terminales.[5,13] Après administration d'adénovirus recombinant, aucune anomalie du comportement n'a été notée chez ces animaux et aucune mortalité n'a été enregistrée.[13] Par contre, plusieurs études ont montré qu'il existait une inflammation pulmonaire dont l'intensité variait avec la dose de virus administrée et qui disparaissait après quelques semaines.[2,3,6,13] L'infiltrat cellulaire était au début essentiellement constitué de neutrophiles, puis de cellules mononucléées, en particulier de lymphocytes et de macrophages vers le 10ème jour. Il n'a été observé ni lésion épithéliale bronchique, bronchiolaire ou parenchymateuse, ni inclusions virales comme celà est décrit dans les infections par les adénovirus sauvages.[3,6,13] Dans une série, une évaluation de la fibrose a été effectuée 28 jours après l'administration du vecteur. Il n'a été retrouvé ni augmentation du collagène ni modification architecturale des territoires péri-bronchiolaires, parenchymateux ou pleuraux.[13]

Chez les primates, babouins ou les singes rhésus, l'effet principal de l'instillation d'adénovirus recombinants est l'apparition d'une inflammation alvéolaire. L'intensité de ces infiltrats inflammatoires parait liée à la concentration du virus. Ces infiltrats s'étendent au delà du territoire où le virus a été instillé traduisant la diffusion du virus en dehors de la zone ciblée. L'analyse microscopique des poumons après autopsie montre une augmentation du nombre de cellules inflammatoires, en particulier des lymphocytes, liée à la dose et qui persiste pendant deux mois chez certains animaux. Ces remaniements inflammatoires se développent en 2 à 3 semaines après l'administration du gène. Ils débutent par un infiltrat lymphocytaire périvasculaire modéré, qui s'étend à l'interstitium pulmonaire chez les sujets recevant les plus fortes doses ($10^9$ à $10^{10}$ pfu). Les tests cliniques ne reflètent pas cette inflammation, sauf pour quelques infiltrats alvéolaires, visibles sur les radiographies pulmonaires et dans les lavages bronchoalvéolaires (BAL).[1,9] Les anomalies radiologiques disparaissent progressivement. Tout au long de ces études, l'aspect physique et le comportement des animaux restent normaux. Enfin, chez tous ces animaux qu'il s'agissent des primates ou des cotton rats, en dehors du poumon, aucune anomalie microscopique n'a été observée.

*Mécanismes impliqués dans les réponses inflammatoires dans les études animales*

Le mécanisme précis de l'inflammation pulmonaire survenant après l'instillation d'adénovirus recombinants n'est pas connu. Le fait que des vecteurs adénoviraux différents comme le AdRSVßGal (contenant l'*Escherichia coli* lac Z) ou l'AdCFTR (contenant l'ADN recombinant du CFTR humain) entraînent le même type d'inflammation, démontre que la réponse inflammatoire est plutôt due aux composants adénoviraux qu'aux gènes transfectés. Il est peu probable que ce soit la lyse cellulaire secondaire à une réplication virale qui soit à l'origine du déclenchement de cette inflammation. Il n'a en effet jamais été retrouvé d'inclusion virale ou de virus sauvage en culture. L'inflammation pulmonaire est plutôt une réponse aux antigènes viraux, probablement liée à l'antigénicité de la protéine de l'enveloppe virale et à une réponse des lymphocytes T cytotoxiques. Le profil et la séquence de ces infiltrats lymphocytaires sont compatibles avec une réponse à des antigènes étrangers. La survenue précoce de polynucléaires neutrophiles correspondrait à une réponse non spécifique de l'hôte. Il a été montré que les cellules épithéliales bronchiques libèrent de l'interleukine-8 (IL-8) après exposition à divers médiateurs comme le tumor necrosis factor (TNF), et que l'IL-8 entraîne un chimiotactisme des polynucléaires neutrophiles, probablement impliqué dans l'inflammation pulmonaire chronique observée dans la CF. Par ailleurs, les adénovirus déficients pour la réplication augmenteraient l'expression des ARN messagers et des protéines de l'IL-8 et de l'ICAM au niveau des cellules A 549 (lignée de cellules pulmonaires).[13]

*Les essais cliniques*

Plusieurs types de protocoles de transfert du gène AdCFTR au niveau de l'épithélium nasal et/ ou bronchique humain ont été menés;[2,3,10,11] le but étant d'évaluer l'efficacité et l'inocuité de ce transfert de gène. Dans une des études, des doses variables d'AdCFTR (jusqu'à $2 \times 10^9$ pfu) ont été administrées dans le nez et 48 heures plus tard dans les poumons de quatre sujets atteints de CF.[2,3] Une expression de l'ARN messager mais également de la protéine CFTR a pu être mise en évidence au niveau de l'épithélium nasal et bronchique. Toutefois, un des sujets a développé un syndrome clinique dans les 12 heures suivant l'administration du virus recombinant, avec fièvre, hypotension, infiltrats pulmonaires plurilobaires bilatéraux, et avec une importante augmentation sérique d'interleukine-6. Par contre, ni adénovirus ni anticorps neutralisants n'ont été mis en évidence. Il s'agissait vraisemblablement d'une réaction inflammatoire pulmonaire directement liée à l'administration de l'adénovirus. Après un mois les symptômes avaient disparu sans séquelles apparentes. Aucun autre agent contaminant n'a été retrouvé. Ce patient avait reçu la plus forte dose d'adénovirus ($2 \times 10^9$ pfu) de l'essai clinique. Il est donc probable que les symptômes observés soient liés à la dose utilisée, mettant ainsi en balance les avantages de la transfection des adénovirus recombinants.

Les expérimentations animales, bien qu'ayant clairement démontré l'existence d'une inflammation pulmonaire secondaire à l'administration d'adénovirus recombinant, ne laissaient pas prévoir l'intensité et la rapidité de l'apparition des symptômes observées dans l'essai humain. Cette différence pourrait être expliquée par les variations d'espèces ou bien encore par l'atteinte pulmonaire chronique toujours observée chez les patients atteints de CF. L'absence de bons modèles animaux pour la CF, en particulier en ce qui concerne la pathologie pulmonaire, limite l'évaluation correcte des transferts de gènes chez l'animal et rend difficile l'extrapolation à la thérapeutique humaine.

Certains auteurs suggèrent que chez l'homme, des études sur la muqueuse nasale pourraient aider à prévoir les problèmes liés aux doses administrées et observés au niveau du poumon.[11] En fait les tests effectués au préalable dans le nez ne permettent pas de prédire ce qui va se passer au niveau du poumon. En effet, bien que le CFTR soit exprimé de façon identique dans les cellules épithéliales du nez et des bronches, son absence chez les sujets atteints de CF a des conséquences différentes, avec des remaniements inflammatoires minimes au niveau de l'épithélium nasal, contrastant avec une inflammation à polynucléaires importante et des modifications dans les pourcentages des différents types de cellules épithéliales observées au niveau de l'épithélium bronchique.[4]

Chez l'animal, l'administration intra-bronchique d'un adénovirus délété pour la région E1 entraîne une inflammation dont l'intensité varie avec la dose et qui disparait sans séquelles apparentes en quelques semaines. L'expérience avec l'instillation est limitée mais l'instillation est usuellement sans conséquences pathologiques, sauf pour un patient. L'importance de ce syndrome ne pouvait être prédit par les expérimentations animales, montrant ainsi l'importance des essais chez l'homme, en particulier dans la CF. En réalité, dans cette affection, le manque de modèle animal, surtout pour la pathologie pulmonaire, et l'importance de l'inflammation sous-jacente compliquent l'évaluation de la toxicité potentielle des vecteurs adénoviraux.

### Références

1. Brody SL, Metzger M, Danel C, Rosenfeld M, Crystal RG. Acute responses of non-human primates to the airway delivery of an adenovirus vector containing the human cystic fibrosis transmembrane conductance regulator cDNA. *Hum Gene Ther* 1994; **5**: 821–836.
2. Crystal RG, Jaffe HA, Brody SL, *et al.* A phase 1 study, in cystic fibrosis patients, of the safety, toxicity, and biological efficacy of a single administration of a replication deficient, recombinant adenovirus carrying the cDNA of the normal cystic fibrosis transmembrane conductance regulator gene in the lung. *Hum Gene Ther* 1995; **6**: 643–666.
3. Crystal RG, McElvaney NG, Rosenfeld MA, *et al.* Administration of an adenovirus containing the human CFTR cDNA to the respiratory tract of individuals with cystic fibrosis. *Nature Genetics* 1994; **8**: 42–51.
4. Danel C, Erzurum S, MacElvaney NG, Crystal RG. Quantitative assessment of human airway epithelial and inflammatory cell populations in cystic fibrosis. *Am J Crit Care Respir Dis* 1996; **153**: 362–368.
5. Mastrangeli A, Danel C, Rosenfeld MA *et al.* Diversity of airway epithelial cell targets for in vivo recombinant adenovirus mediated gene transfer. *J Clin Invest* 1993; **91**: 225–234.
6. Prince GA, Porter DD, Bennett Jenson A, *et al.* Pathogenesis of adenovirus type 5 pneumonia in cotton rats (*Sigmodon hispidus*). *J Vir*, 1993; **67**: 101–111.
7. Rosenfeld MA, Yoshimura K, Trapnell BC, *et al. In vivo* transfer of the human cystic fibrosis transmembrane conductance regulator gene to the airway epithelium. Cell, 1992; **68**: 143–155.
8. Rosenfeld MA, Chu CS, Seth P, *et al.* Gene transfer to freshly isolated human respiratory epithelial cells in vitro using a replication deficient adenovirus containing the human transmembranne conductance regulator cDNA. *Hum Gene Ther* 1994; **5**: 331–342.
9. Simon RH, Engelhard JF, Yang Y, *et al.* Adenovirus-mediated transfer of the CFTR gene to lung of nonhuman primates: toxicity study. *Hum Gene Ther* 1993; **4**: 771–780.
10. Welsh, MJ, Smith AE, Zabner J, *et al.* Cystic fibrosis gene therapy using an adenovirus vector; in vivo safety and efficacy in nasal epithelium. *Hum Gene Ther* 1994; **5**: 209–219.
11. Wilson JJ, Engelhardt JF, Grossman R, Simon RH, Yang Y. Gene therapy of cystic fibrosis lung disease using E1 deleted adenoviruses: a phase 1 trial *Hum Gene Ther* 1994; **5**: 501–519.
12. Wilson J. Gene Therapy for cystic fibrosis: challenges and futures directions. *J Clin Invest* 1995, **96**: 2547–2554.
13. Yei S, Mittereder N, Wert S, Whitsett JA, Wilmott RW, Trapnell BC. *In vivo* evaluation of the safety of adenovirus-mediated transfer of the human cystic fibrosis transmembrane conductance regulator cDNA to the lung. *Hum Gene Ther* 1994; **5**: 731–744.
